# Systematic analysis of gene expression alterations and clinical outcomes of adenylate cyclase-associated protein in cancer

**DOI:** 10.18632/oncotarget.16111

**Published:** 2017-03-10

**Authors:** Shuanshuan Xie, Changxing Shen, Min Tan, Ming Li, Xiaolian Song, Changhui Wang

**Affiliations:** ^1^ Department of Respiratory Medicine, Shanghai Tenth People's Hospital, Tongji University, Shanghai 200072, China

**Keywords:** adenylate cyclase-associated protein, copy number alteration, mutation, overall survival

## Abstract

Adenylate Cyclase-associated protein (CAP) is an evolutionarily conserved protein that regulates actin dynamics. Our previous study indicates that CAP1 is overexpressed in NSCLC tissues and correlated with poor clinical outcomes, but CAP1 in HeLa cells actually inhibited migration and invasion, the role of CAP was discrepancy in different cancer types. The present study aims to determine whether CAP can serve as a prognostic marker in human cancers. The CAP expression was assessed using Oncomine database to determine the gene alteration during carcinogenesis, the copy number alteration, or mutations of CAP using cBioPortal, International Cancer Genome Consortium, and Tumorscape database investigated, and the association between CAP expression and the survival of cancer patient using Kaplan-Meier plotter and PrognoScan database evaluated. Therefore, the functional correlation between CAP expression and cancer phenotypes can be established; wherein CAP might serve as a diagnostic marker or therapeutic target for certain types of cancers.

## INTRODUCTION

Cancer is the most important reason for death globally, and the cancer-related morbidity and mortality rate is anticipated to increase over the next couple years. On the basis of the WHO (World Health Organization), if the total cancer incidence remains relatively constant, by 2030, the number of new cancer cases will more than doubled to today [1]. Irreparable structural mutations in cells are the main cause of human cancer. These mutations can alter the DNA copy number and function of the gene at very specific genomic locations [2, 3]. Pollack et al. established that CNAs uncover all gene expression, which could be a critical element in the tumor development [4]. Identifying copy number alterations can state a method for linking CNA with disease phenotype [5]. This study can provide theoretical basis for clinicians and researchers, and finds out a new signaling pathway or biomarker in cancer which is helpful to develop the therapeutic approach for early intervention in preventing cancer.

Cell proliferation and elevated invasiveness are the two most prominent hallmarks of cancer cells that result in the majority of cancer patients’ deaths [6]. The actin cytoskeleton is essential for many cellular functions including cell migration. Several pieces of evidence have shown that the actin-regulating protein is involving in the cell motility and invasiveness of human cancers. Adenylate cyclase-associated protein (CAP) is one of the major actin-regulating proteins in cancers.

CAP was first found in yeast. It is an actin monomer-binding protein coded by the *CAP* gene and has been observed to involve in cell motility and progress of particular kinds of cancers. The two isoforms of CAPs identified in mammals are CAP1 and 2. CAP1 exists in almost all tissues and cells, But, CAP2 is expressed in specific tissues and cells. Recent studies, including those from our group, have established that mammalian CAP1 is overexpressed in NSCLC tissues and correlated with poor clinical outcomes [7]. On the other hand, CAP2 has been found to be overexpressed in melanoma and hepatocellular carcinoma [8–9]. Knockdown of CAP1 leads to reduced cell motility in lung and pancreatic cancer [7, 10]. However, depletion of CAP1 in HeLa cells and breast cancer cells substantially stimulates the migration and invasion [11, 12]. These results indicate that CAP may either play oncogenic or anti-oncogenic function hinging on cancer types. Nevertheless, the exact role of CAP expression remains elusive and controversial based on the conflicting evidence.

To explore the character of CAP members in cancers, Oncomine platform assesses the gene expression of cancers by nearly 90,000 microarray experiments (http://www.oncomine.com) [13, 14]. Furthermore, the survival of cancer patients was assessed by Kaplan-Meier plotter and PrognoScan database [15, 16]. The co-expression data revealed the biological function and provided insight into the potential underlying mechanism. The gene ontology enrichment by STRING (http://string-db.org) is able to discover the function and regulatory mechanism of genes [17, 18]. The present aimed to determine if the CNAs of the CAP axis correlated with aggressive cancer sub-types, based on the cBioPortal and Tumorscape [19–22] (Table 1). The first study showing the role of CAP in cancers. Therefore, detailed analyses of CAP1 and CAP2 have been described below.

**Table 1:** Main characteristic of the selected oncogenomic portals

database	Data source	Sites of analyzed cancer 1	Oncogenomic data	link
**Oncomine**	TCGA, Cancer data from literature	Bd; Br; Bra; Cer; Clr; Eso; HN; Kd; Lng; Lvr; Lymph; Ov; Pnc; also: cancer cell lines	Drug Sensitivity, Cancer histology, clinical outcome, tissue, pathology, subtype, molecular subtype, patient treatment response	https://www.oncomine.org [13, 14]
**Kaplan-Meier plotter**	Cancer data from literature	Br, Ov; Lng; GIST;	survival analyses	http://kmplot.com/analysis [15]
**Prognoscan**	Cancer data from literature	Bd; Bld; Br; Bra; Clr; EA; Eso; HN; Kd; Lng; Lymph; Ov; Prst; Sk; ST;	survival analyses	http://www.abren.net/PrognoScan/[16]
**STRING**	Protein, gene from literature	Gene, gene from literature	Structure	http://string-db.org [17, 18]
**cBioPortal**	AMC, BCCRC, BGI, British Columbia, Broad, Broad/ Cornell, CCLE, CLCGP, Genentech, ICGC, JHU, Michigan, MKSCC, MKSCC/ Broad, NCCS, NUS, PCGP, Pfizer UHK, Riken, Sanger, Singapore, TCGA, TSP, UTokyo, Yale	ACC; Bd; Bld; Br; Bra; Chl; Clr; Eso; HN; Kd; Lng; Lvr; Lymph; MM; Npx; Ov; Pnc; Prst; Sk; ST; Stc; Thr; Utr; also: cancer cell lines	mutations, putative copy number alterations; mRNA expression, protein/ phosphoprotein level; survival analyses	http://www.cbioportal.org/ [19, 20]
**Tumorscape**	Broad Institute	Bd; Bld; Br; Bra; Clr; Eso; GIST; HN; Htp; Kd; Lng; Lvr; Lymph; Msh; Ov; Pnc; Prst; Sk; ST; Stc; Swn; Thr; Utr; also in: cancer cell lines	copy number alterations	http://www.broadinstitute.org/tumorscape/ [21, 22]
**ICGC**	ICGC, TCGA, TARGET	ACC; Bd; Bld; Br; Bra; Chl; Clr; Eso; HN; Kd; Lng; Lvr; Lymph; MM; Npx; Ov; Pnc; Prst; Sk; ST; Stc; Thr; Utr; also: cancer cell lines	simple somatic mutations, copy number somatic alterations, structural somatic mutations, simple germline variants, DNA methylation, gene/ protein expression, miRNA expression, exon junction; epidemiological and clinical data	https://dcc.icgc.org.[73, 74]

1 List of abbrieviations of cancer sites. In the brackets there are exemplary cancer subtypes included in the portals. ACC – adenoid cystic carcinoma; Bd – bladder; Bld – blood; Bo – bone; Br – breast; Bra – brain; Chl – cholangiocarcinoma; Clr – colorectal; Col – colon; EA – eye and adnexa; EG - endocrine glands; Eso – esophagus; GIST – gastrointestinal; HN – head and neck; Htp – hematopoietic; Kd – kidney; Lng – lung; Lvr – liver and biliary tract; Lymph – Lymphoma; Msh – mesothelioma; Mth – mouth; Nb – neuroblastoma; Npx – nasopharynx; Ov – ovary; Pan – pancancer; Pnc – pancreas; Pnx – pharynx; Prc/Prn - pheochromocytoma and paraganglioma; Prst – prostate; Rc – rectum; Sk – skin; ST – soft tissues; Stc – stomach; Swn – schwannoma; Thm – thymus; Thr – thyroid; Tst – testis; Utr – uterine (cerxix and corpus).

CAP1 may play a potential oncogenic role in pancreatic cancer and tumor suppressor in breast cancer. Also, the relationship between CAP1 expression levels and patient survival was observed. The co-expression analysis revealed that CAP1 was coexpressed with TUBA1B in pancreatic and head-neck cancer, as well as with CFL1, CFL2, DSTN, ACTB, ACTG1, and ROBO1, according to the STRING analysis. High CAP2 expression showed a relatively good prognosis in breast cancer, whereas poor prognosis in gastric and ovarian cancers. CAP2 was coexpressed with TP53BP2 and ENA/VASP in liver cancer. The cBioPortal and Tumorscape analysis found that frequency of 40.2–20% with the rank order mainly in prostate cancer with CAP1, CAP2 alteration ranged from 38.3–19.8%. The CAP mutations occur in a hotspot in the CAP N domain. Totally, the different subtype of CAP is involves in different cancer types. CAP-coexpressed molecules may be established further to elucidate the function of CAP members in the specific type of cancer. Together, these results provide additional support for CAP as a biomarker and a target for cancer therapy.

## RESULTS

**Figure 1:**
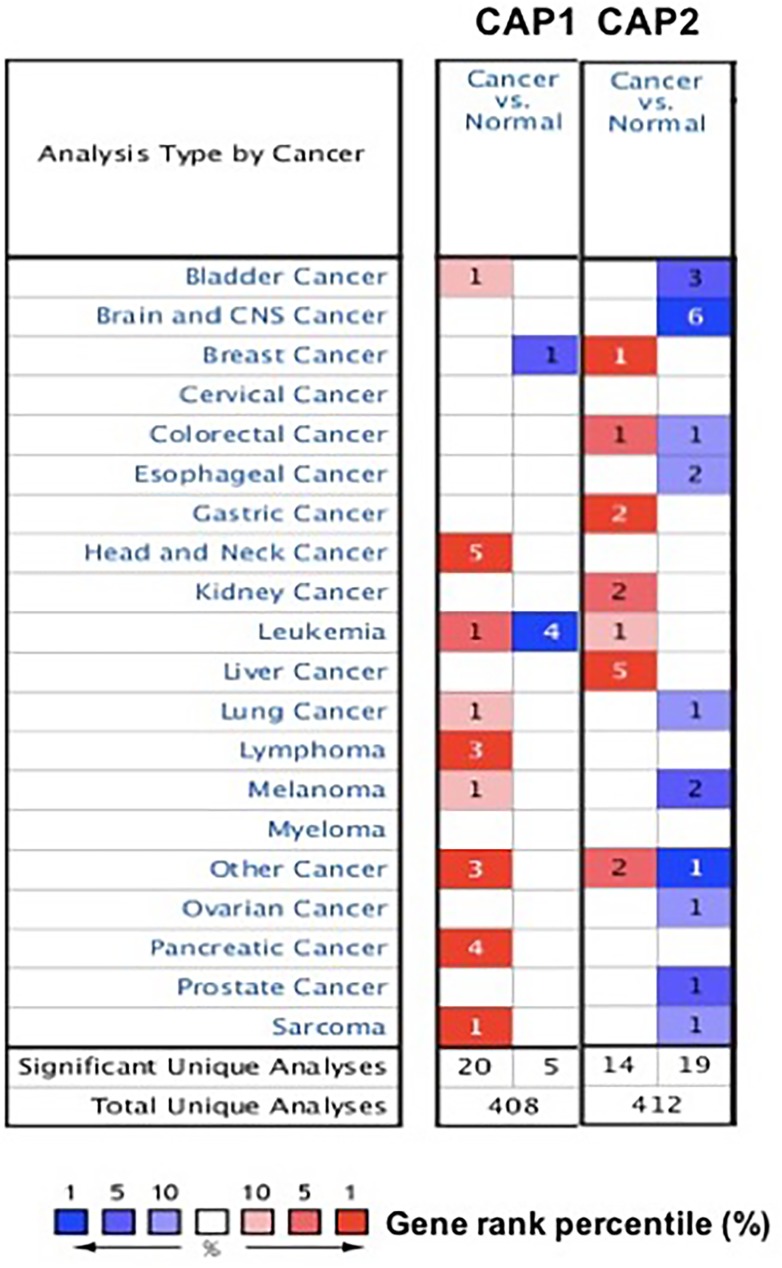
CAP mRNA expression in various cancer types The comparison indicated the number of datasets with CAP mRNA overexpression (right column, red) and under expression (left column, blue) in cancer versus normal tissue. The threshold was designed with following parameters: *p*-value of 1E-4, fold change of 2, and gene ranking of 10%.

To explore the role of CAP in cancers. The expression of CAP was analyzed between tumor and normal tissues using the Oncomine database. The threshold was designated according to the following values: *p*-value 1E-4, fold change 2, and top gene ranks 10%. The CAP was over-expressed in certain types of cancers and lower in others as compared to that of the normal tissue. These results indicate that CAP may work either oncogenic or anti-oncogenic function according to the cancer types (Figure 1). Thus, detailed analyses of CAP1 and CAP2 were described below.

### CAP transcript expression by cancer type

**Figure 2:**
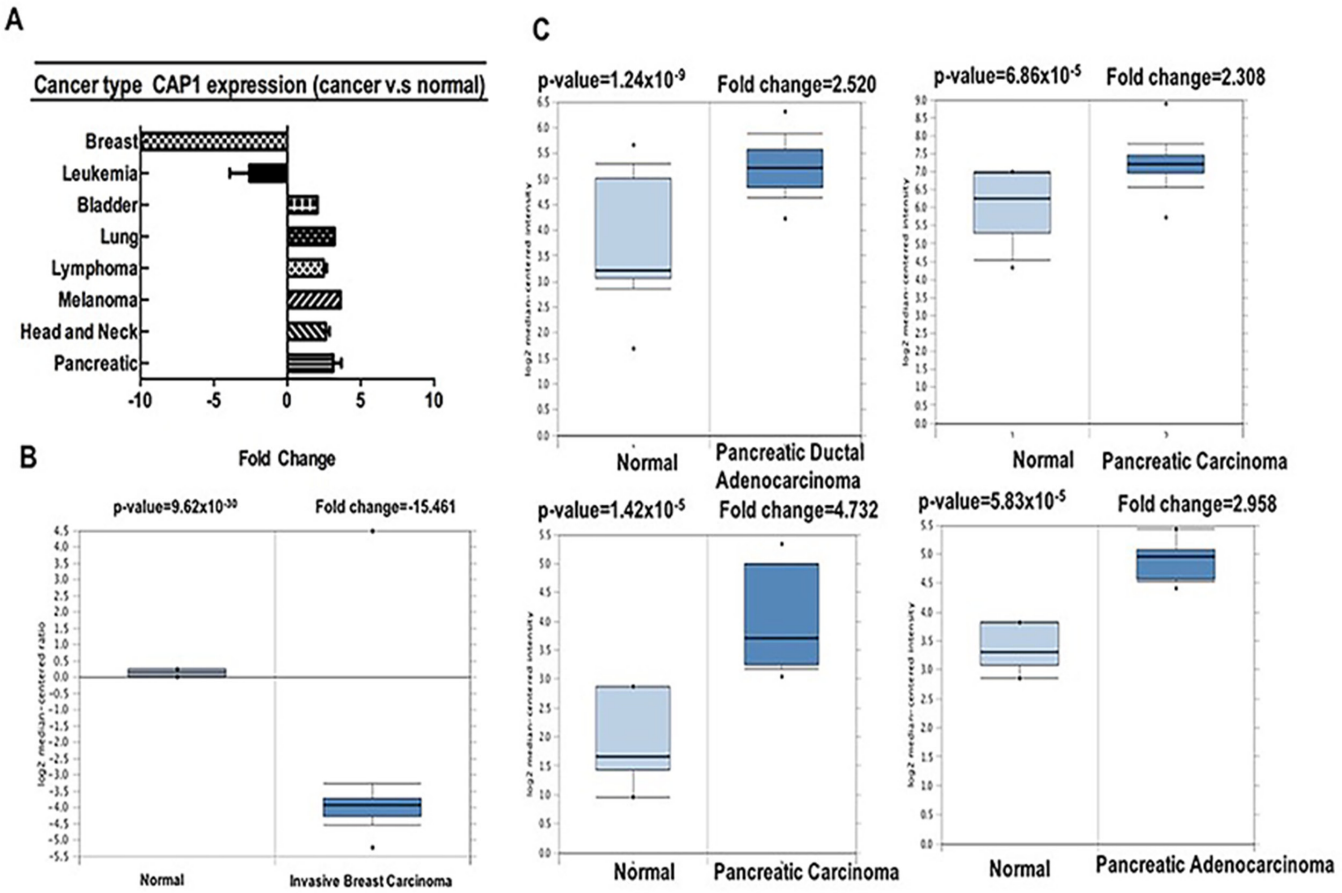
CAP1 analysis in different cancer types (Oncomine database) The box plot comparing specific CAP1 expression in normal (left plot) and cancer tissue (right plot) was derived from Oncomine database. The fold change of CAP1 in various types of cancers was identified from our analyses in Table 1 and expressed as the forest plot (**A**). The analysis was shown in breast carcinoma relative to normal breast (**B**), in pancreatic carcinoma relative to normal pancreatic (**C**).

CAP has been shown as an actin-regulatory protein. Mammals harbor two CAP isoforms, CAP1 and CAP2 [23, 24]. Depletion of CAP1 reduces actin dynamics, cell motility and cell invasion [11, 23, 24]. Our analysis revealed that CAP1 was over-expressed in bladder, lung, lymphoma, melanoma, head-neck, pancreatic cancers, but was under-expressed in breast and leukemia cancers as compared to that in normal tissue (Table 2, Figure 2, Supplementary Figures 1–3) [25–39]. These data are in agreement with the previously published reports on CAP1 expression. For instance, our study indicated that CAP1 is highly expressed in lung cancer [7] (Supplementary Figure 1B), elevated in pancreatic cancer [10] (Figure 2C).

**Table 2:** CAP1 expression in cancers

Cancer	cancer subtype	*p* −value	fold change	rank (%)	sample	reference
**Bladder**	Infiltrating Bladder Urothelial Carcinoma	7.84E5	2.046	10	27	[25]
**Breast**	Invasive Breast Carcinoma Stroma	9.62E–30	−15.461	2	59	[26]
**Head and Neck**	Tongue Squamous Carcinoma	1.68E–12	3.333	1	57	[27]
	Tonsillar Carcinoma	4.23E–5	2.1036	1	10	[28]
	Tongue Carcinoma	4.3E–7	2.439	2	19	[28]
	Oropharyngeal Carcinoma	7.91E–5	2.1	3	10	[28]
	Tongue Squamous Carcinoma	1.37E–11	3.095	2	57	[29]
**Leukemia**	Chronic lymphocytic leukemia	9.13E–6	2.212	5	111	[30]
	Acute myeloid leukemia	3.79E–7	−5.989	1	31	[31]
	T−cell acute lymphoblastic	8.00E–9	−3.731	2	17	[32]
	Acute myeloid leukemia	2.59E–7	−2.198	5	144	[32]
	T−cell acute lymphoblastic	1.25E–7	−3.135	9	93	[32]
**Lung**	Squamous cell lung Carcinoma	4.89E–13	3.2211	1	26	[29]
**Lymphoma**	Anaplastic larger cell lymphoma	9.81E–5	2.140	1	27	[33]
	Angioimmunoblastic T−cell lymphoma	9.82E–10	2.874	1	26	[33]
	Unspecified peripheral T−cell lymphoma	2.79E–13	2.391	3	48	[33]
**Melanoma**	Cutaneous Melanoma	2.31E–5	3.629	8	52	[34]
**Pancreatic**	Pancreatic carcinoma	1.42E–5	4.732	1	17	[35]
	Pancreatic adenocarcinoma	5.83E–5	2.958	4	15	[36]
	Pancreatic carcinoma	6.86E–5	2.308	8	52	[37]
	Pancreatic ductal adenocarcinoma	1.24E–9	2.520	5	78	[38]
**Other**	Embryonal carcinoma	1.16E–7	2.689	5	20	[39]
	Seminoma, NOS	5.64E–9	2.112	2	18	[39]
	Teratoma, NOS	1.60E–8	2.067	3	20	[39]

### Genetic alterations of CAP and overall survival (OS)

Kaplan-Meier plotter analysis showed the relationship between the overexpression of CAP1 and overall high survival rates in lung cancer. Contrastingly, breast and ovarian cancers showed the relationship between overexpression of CAP1 and overall low survival rates (Supplementary Figure 4).

**Figure 3:**
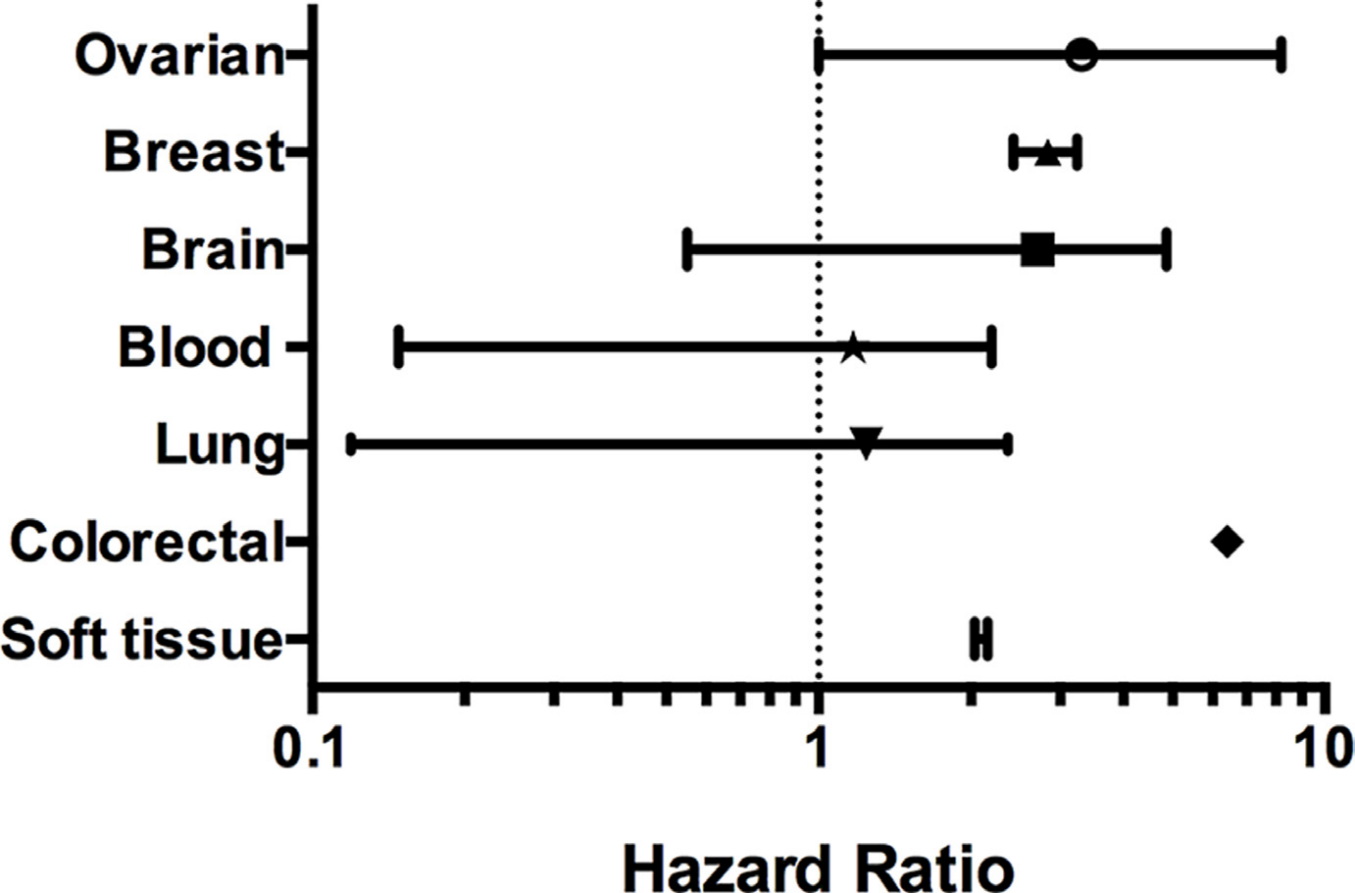
CAP1 genes in different cancer types (PrognoScan database) The statistically significant hazard ratio in various types of cancers was identified from our analyses in Table 2 and expressed as the forest plot. The analysis of survival curve was identified as the threshold of cox *p*-value < 0.05.

**Figure 4:**
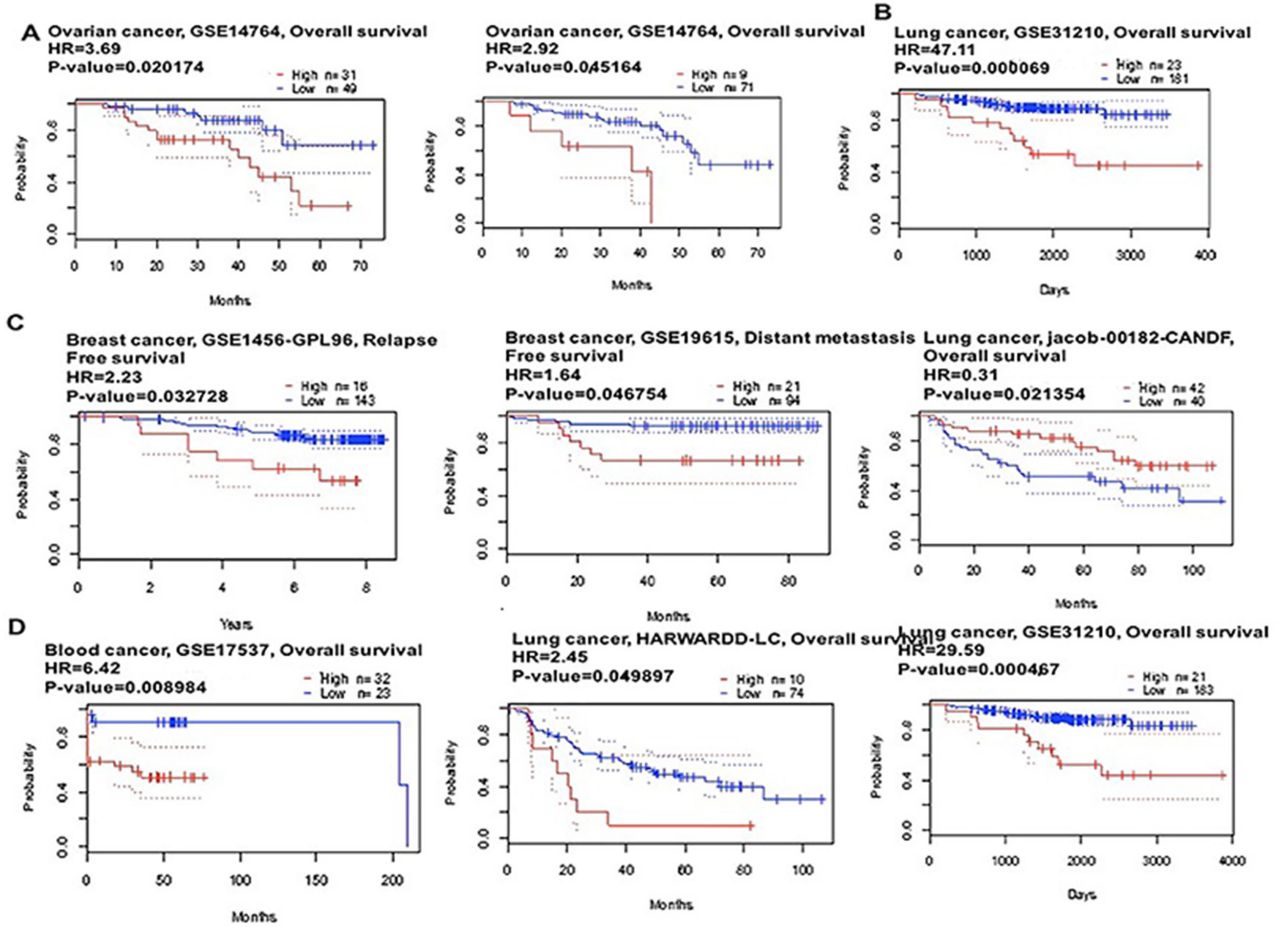
CAP1 genes in Ovarian, Lung, Breast and Blood cancer types (PrognoScan database) The survival curve comparing the patient with high (red) and low (black) expression was plotted from PrognoScan database. The survival curve comparing the patient with high (red) and low (black) expression in ovarian cancer (**A**), lung cancer (**B**), breast cancer (**C**) and blood cancer (**D**) was plotted from PrognoScan database as the threshold of cox *p*-value < 0.05.

The prognostic value of CAP1 expression was reported by PrognoScan database (Figure 3, Table 4). The poor prognosis in breast and ovarian cancer patients with higher CAP1 expression was in line with the data from Kaplan-Meier plotter analysis (Figure 4A, 4C, Supplementary Figure 4). The Oncomine, PrognoScan and Kaplan-Meier plotter data showed the oncogenic role of CAP1 in breast, ovarian, blood, and brain cancer; however that in lung cancer is not clear (Supplementary Figure 4A, 4B). Thus, to evaluate the oncogenic or tumor suppressor role of CAP1 in lung cancer, our previous study demonstrated that the protein expression was significantly higher in NSCLC tissues compared with their matched normal lung tissues. In addition, the expression of CAP1 in tumor tissues was significantly associated with a tumor, lymph node metastasis, and TNM stage in NSCLC patients; CAP1 was highly expressed in lung cancer with brain metastasis as compared to other metastatic groups (bone metastasis and visceral metastasis). Kaplan-Meier analysis showed that the OS rate in NSCLC patients with high CAP1 levels was significantly lower than that in those with low CAP1 levels [7, 40].

**Table 3:** The association of CAP1 expression and the survival in cancer patients

Cancer type	*N*	COX *P*-VALUE	HR	ENDPOINT	DATASET	PROBE ID
Blood	79	4.58 E–02	1.76	Overall Survival	GSE12417-GPL570	213798_s_at
	79	3.46 E–02	1.83	Overall Survival	GSE12417-GPL570	200625_s_at
	34	2.6 E–02	3.15	Overall Survival	GSE8970	200625_s_at
	158	8.44 E–03	0.49	Overall Survival	GSE4470	213798_s_at
	158	3.87 E–03	0.46	Overall Survival	GSE4475	200625_s_at
	53	4.49 E–02	0.25	Overall Survival	E-TABN-346	200625_s_at
	53	3.40 E–02	0.26	Overall Survival	E-TABN-346	200625_s_at
**Brain**	50	1.05E–02	1.74	Overall Survival	MGH-glioma	935_at
	74	4.32E–02	2.96	Overall Survival	GSE4412-GPL96	200625_s_at
	74	2.01E–02	3.42	Overall Survival	GSE4412-GPL96	213798_s_at
**Breast**	115	4.68E–02	5.13	Distant Metastasis Free Survival	GSE19615	213798_s_at
	159	8.93E–03	1.78	Disease specific Survival	GSE1456-GPL96	213798_s_at
	159	1.94E–02	3.36	Relapse Free Survival	GSE1456-GPL96	200625_s_at
	159	2.07E–02	4.17	Disease specific Survival	GSE1456-GPL96	200625_s_at
	159	3.27E–02	2.23	Relapse Free Survival	GSE1456-GPL96	213798_s_at
	236	4.34E–02	2.9	Disease specific Survival	GSE1456-GPL96	200625_s_at
	236	1.40E–02	3.35	Disease specific Survival	GSE1456-GPL96	213798_s_at
	249	2.45E–02	2.39	Disease specific Survival	GSE1456-GPL96	213798_s_at
	249	2.13E–02	2.65	Disease specific Survival	GSE1456-GPL96	200625_s_at
**Colorectal**	55	8.98E–03	6.42	Disease free Survival	GSE17537	213798_s_at
**Lung**	82	2.14E–03	0.31	Overall Survival	Jacob-00182-CANDF	213798_s_at
	84	4.99E–02	2.45	Overall Survival	HARVAD-LC	935_at
	204	4.67E–05	29.59	Overall Survival	GSE31210	213798_s_at
	204	1.21E–05	17.93	Relapse free Survival	GSE31210	213798_s_at
	204	6.9E–05	47.11	Overall Survival	GSE31210	200625_s_at
	204	1E–05	34.9	Relapse free Survival	GSE31210	200625_s_at
	138	8.4E–05	2.23	Relapse free Survival	GSE8894	213798_s_at
	138	2.39E–03	1.94	Relapse free Survival	GSE8894	200625_s_at
**Overian**						
	80	4.51E–02	2.92	Overall Survival	GSE14764	200625_s_at
	80	2.02E–02	3.69	Overall Survival	GSE14764	213798_s_at
**Soft tissue cancer**	140	2.22E–02	2.1	Distant Recurrence Free Survival	GSE130929	200625_s_at
	140	1.83E–02	2.09	Distant Recurrence Free Survival	GSE130929	213798_s_at

**Table 4:** CAP2 expression in cancers

Cancer	cancer subtype	*p*-value	fold change	rank (%)	sample	reference
**Bladder**	Superficial Bladder cancer	2E–15	−7.061	3	76	[41]
	Superficial Bladder cancer	2.79E–15	−2.41	4	194	[41]
	Infiltrating Bladder Urothelial Carcinoma	9.95E–15	−4.549	5	129	[42]
**Brain**	Glioblastoma	4.70E–17	−7.893	1	31	[43]
	Brain Glioblastoma	1.87E–22	−5.558	1	552	TCGA
	Glioblastoma	8.42E–12	−3.522	2	25	[42]
	Glioblastoma	2.90E–6	−4.275	3	84	[44]
	Oligodendroglioma	3.58E–7	−3.842	6	42	[45]
**Breast**	Invasive Ductal Breast Carcinoma Epithelia	7.92E–6	2.074	1	23	[46]
**Colorectal**	Colon Carcinoma	5.13E–8	2.038	5	15	[47]
	Colorectal Adenocarcinoma	1.30E–6	−2.125	7	69	[47]
**Esophageal**	Barrett's Esophagus	7.38E–9	−2.728	8	43	[48]
	Esophageal Adenocarcinoma	1.26E–11	−2.124	9	103	[48]
**Gastric**	Diffuse Gastric Adenocarcinoma	1.73E–5	4.120	1	37	[49]
	Gastric Mixed Adenocarcinoma	1.43E–6	3.077	2	35	[49]
**Kidney**	Clear Cell Renal Cell Carcinoma	1.67E–6	3.098	5	31	[50]
	Papillary Renal Cell Carcinoma	4.18E–5	2.459	3	5	[50]
**Leukemia**	Pro-B Acute Lymphoblastic Leukemia	8.93E–16	2.209	8	144	[51]
**Liver**	Hepatocellular Carcinoma	3.27E–99	5.790	1	445	[52]
	Hepatocellular Carcinoma	2.26E–13	8.569	1	45	[53]
	Liver Cell Dysplasia	9.81E–5	2.140	1	27	[53]
	Hepatocellular Carcinoma	1.41E–28	3.526	1	179	[54]
	Hepatocellular Carcinoma	2.92E–10	4.254	1	43	[52]
**Lung**	Squamous Cell Lung Carcinoma	1.27E–6	−4.154	8	62	[29]
**Melanoma**	Cutaneous Melanoma	2.03E–9	3.743	3	52	[55]
	Benign Melanocytic Skin Nevus	3.50E–5	−2.859	3	25	[55]
**Ovarian**	Ovarian Serous Adenocarcinoma	1.44E–8	−3.384	7	53	[56]
**Prostate**	Prostate Carcinoma	5.86E–6	−2.238	2	34	[57]
**sarcoma**	Myxofibrosarcoma	5.56E–5	−3.711	10	40	[58]
**Other**	Teratoma, NOS	1.16E–7	2.689	5	20	[59]
	Yolk Sac Tumor, NOS	3.18E–6	2.342	4	15	[59]
	Vulvar Intraepithelial Neoplasia	2.24E–6	−2.809	1	19	[60]

**Figure 5:**
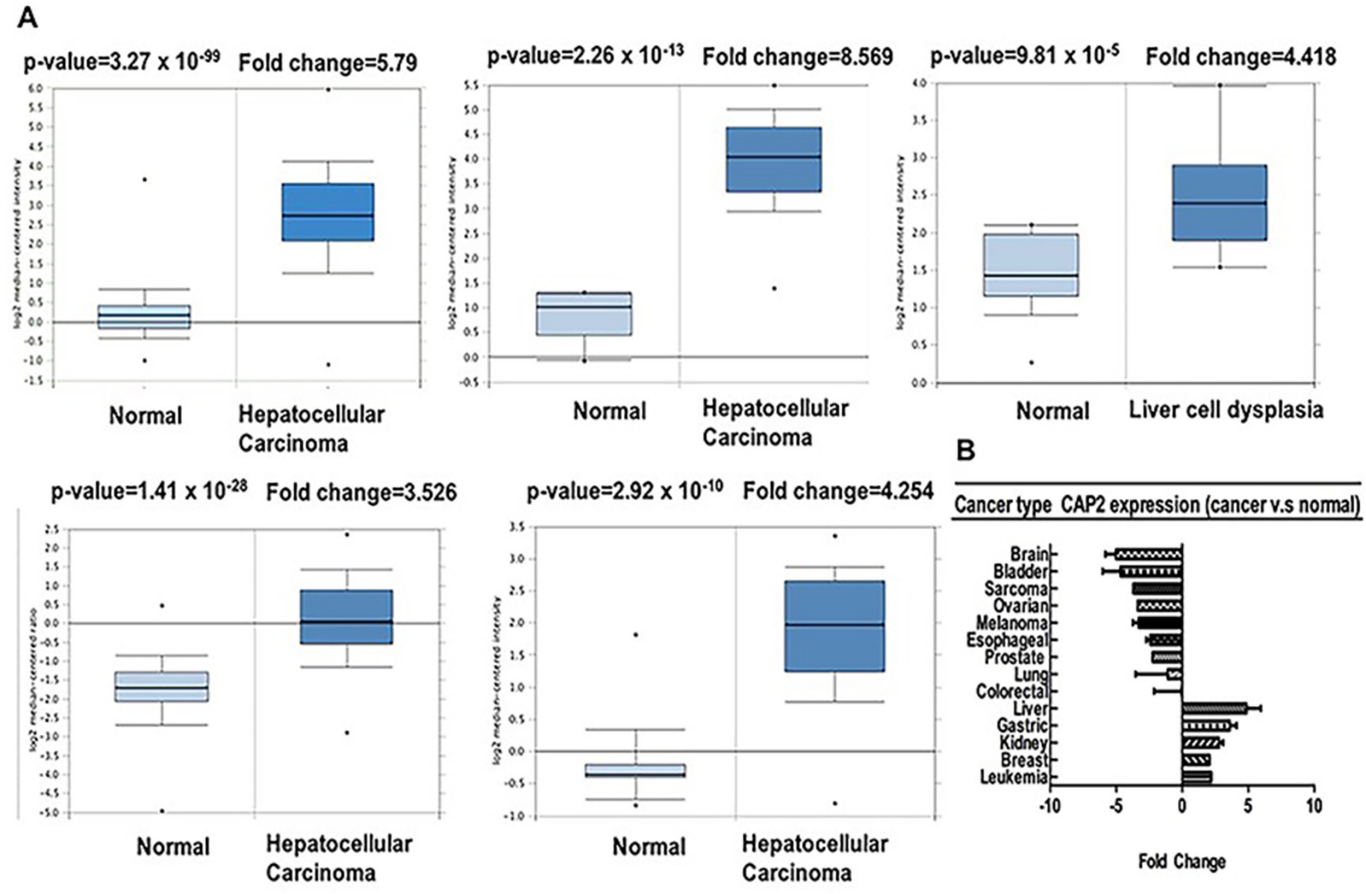
CAP2 analysis in different cancer types (Oncomine database) The box plot comparing specific CAP2 expression in normal (left plot) and cancer tissue (right plot) was derived from Oncomine database. The fold change of CAP2 in various types of cancers was identified from our analyses in Table 3 and expressed as the forest plot (**B**). The analysis was shown in Liver carcinoma relative to normal liver (**A**).

Furthermore, we used Oncomine to confirm the CAP2 expression in different types of cancers (Table 3) [41–60]. CAP2 was upregulated in leukemia, gastric, breast, kidney, and liver cancer, whereas decreased in the bladder, brain, lung, prostate, oesophagus, and ovarian cancer, as well as sarcoma and melanoma (Figure 5A, Supplementary Figures 5–7). These data are in agreement with the previously published reports on CAP2 expression. For instance, CAP2 has been found to be overexpressed in hepatocellular carcinoma [8, 9] (Figure 5B).

**Figure 6:**
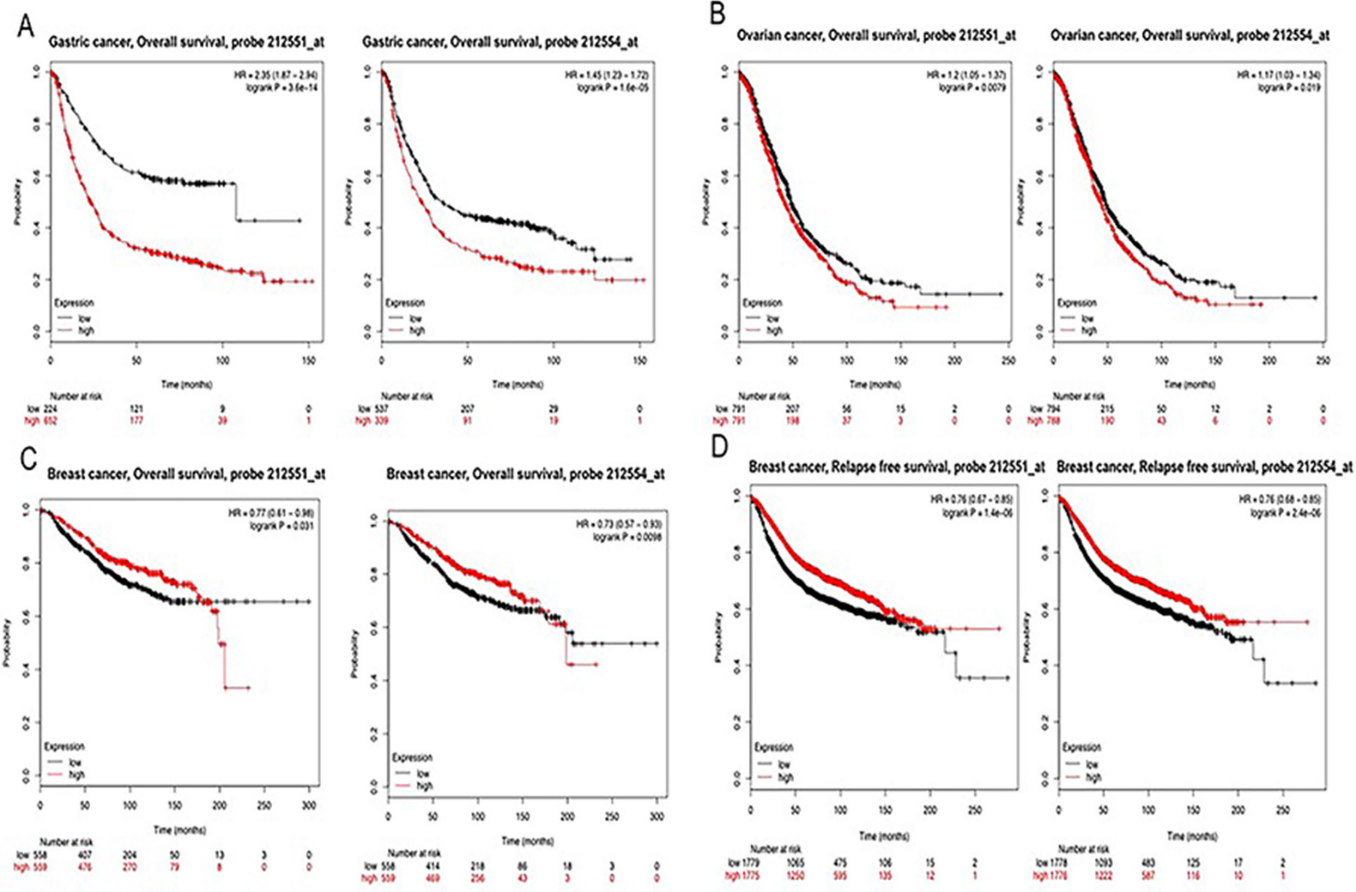
CAP2 genes in Breast, Ovarian and Gastric cancer (Kaplan-Meier Plotter) The survival curve comparing the patient with high (red) and low (black) expression in breast, ovarian and gastric cancer was plotted from Kaplan-Meier plotter database.

**Figure 7:**
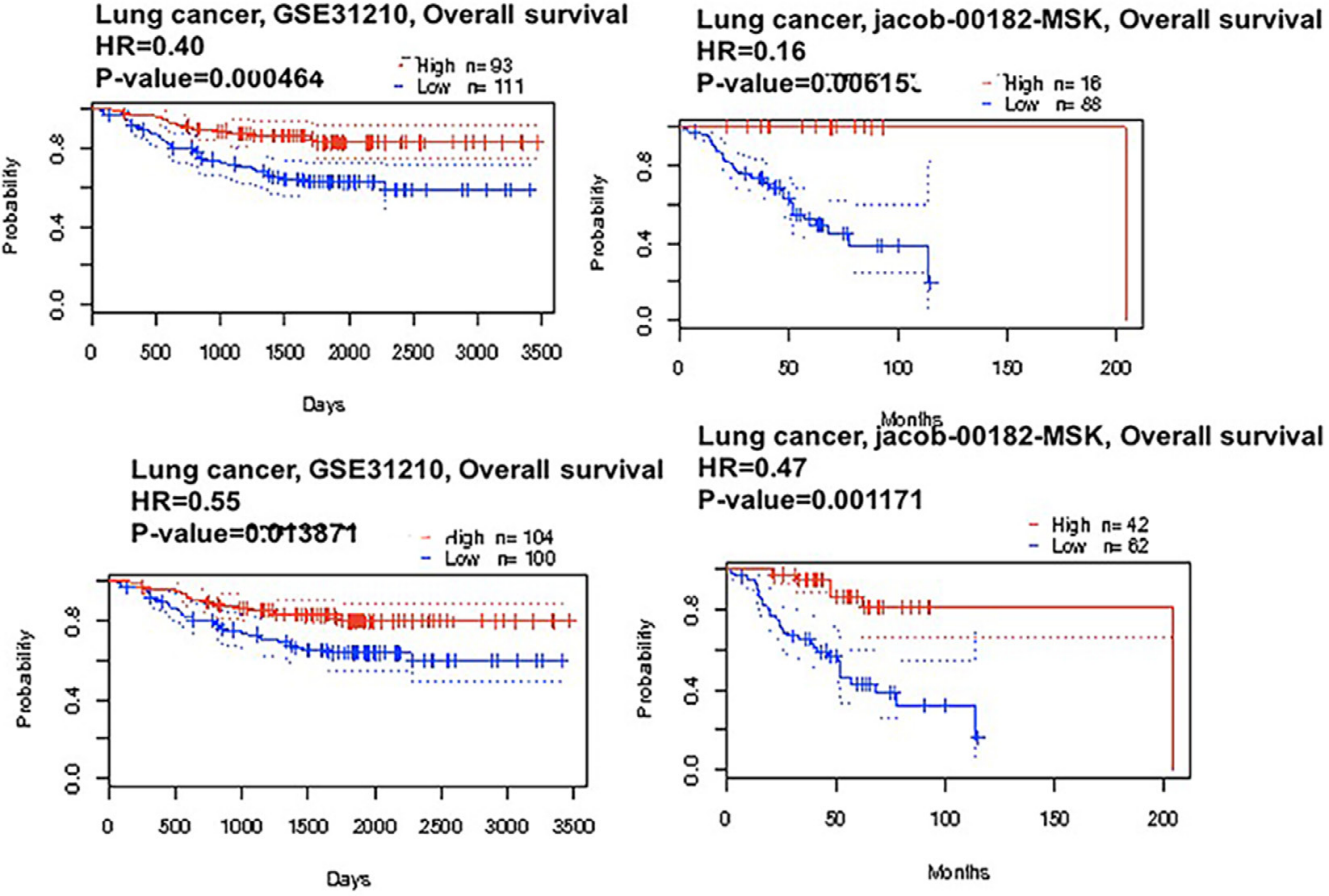
CAP2 genes in Lung cancer (PrognoScan database) The survival curve comparing the patient with high (red) and low (black) expression in lung cancer were plotted from PrognoScan database as the threshold of cox *p*-value < 0.05.

**Figure 8:**
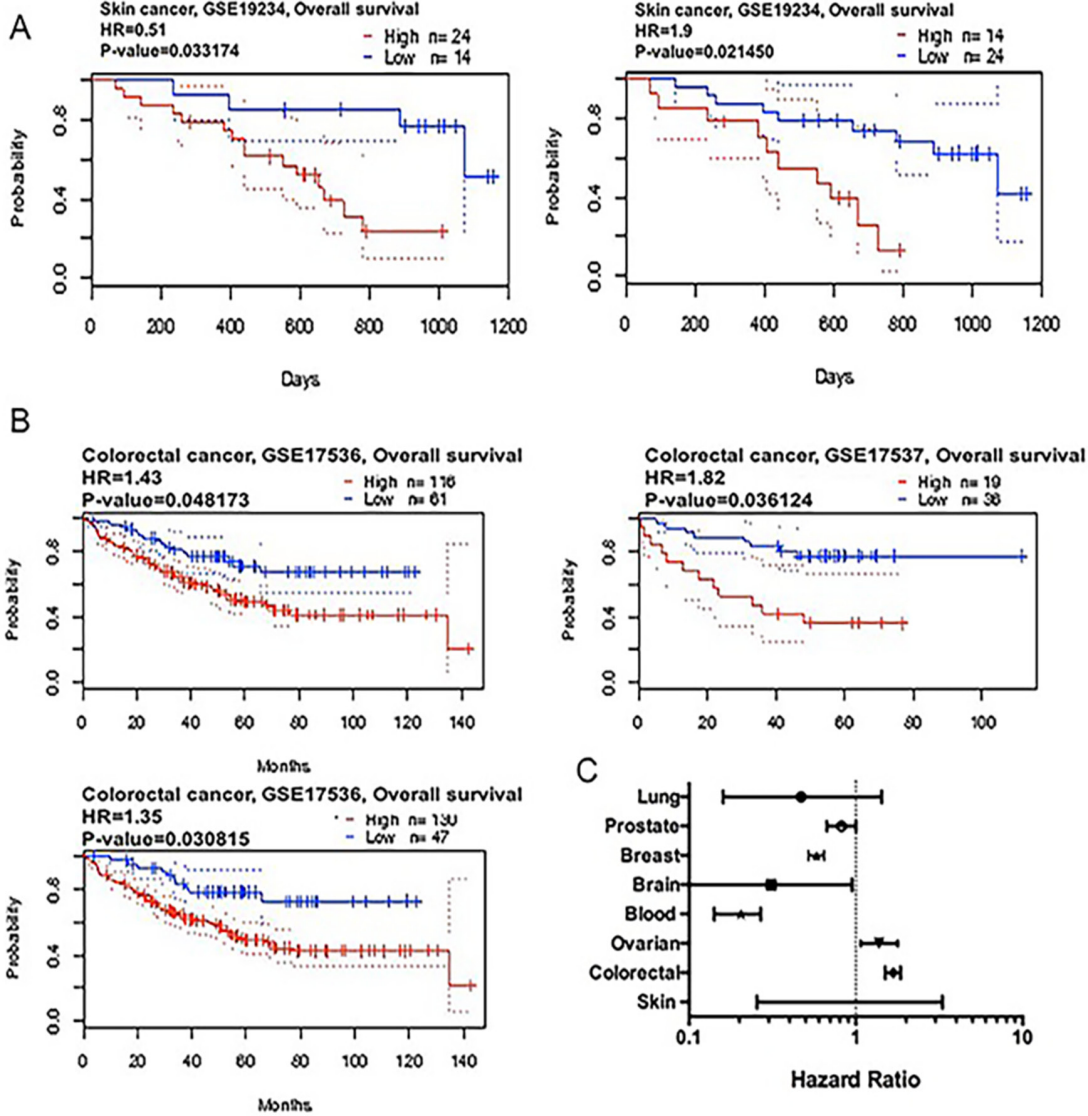
CAP2 genes in Skin and Colon cancer (PrognoScan database) The survival curve comparing the patient with high (red) and low (black) expression in skin (**A**) and colon cancer (**B**) were plotted from PrognoScan database as the threshold of cox *p*-value < 0.05. The statistically significant hazard ratio in various types of cancers was identified from our analyses in Table 4 and expressed as the forest plot (**C**).

We applied the Kaplan-Meier plotter to identify the OS of breast, ovarian, and gastric cancer patients. The results showed that CAP2 was associated with poor survival in gastric and ovarian cancers, but with better survival in breast cancer (Figures 6, 7). The prognostic value of CAP2 was reported by PrognoScan database (Figure 8C, Table 5). The improved prognosis was observed in the brain, blood, and prostate cancer patients, whereas poor prognosis in the skin and colorectal cancers (Figure 8A–8B, Supplementary Figure 8).

**Table 5:** The association of CAP2 expression and the survival in cancer patients

Cancer type	*N*	COX *P*-VALUE	HR	ENDPOINT	DATASET	PROBE ID
**Blood**						
	34	9.83 E–3	0.21	Overall survival	GSE8970	212551_at
	158	6.49 E–3	0.2	Overall survival	GSE4475	212554_at
**Brain**						
	67	4.07 E–2	0.31	Overall survival	GSE16581	HG-U133_plus_2
**Breast**						
	159	8.04 E–3	0.57	Disease specific survival	GSE1456-GPL96	212554_at
	159	1.13 E–2	0.55	Relapse free survival	GSE1456-GPL96	212551_at
	159	4.23E–2	0.57	Disease specific survival	GSE1456-GPL96	212551_at
	159	1.93 E–2	0.64	Relapse free survival	GSE1456-GPL96	212554_at
**Colorectal**						
	177	4.82 E–2	1.43	Overall survival	GSE17536	212551_at
	177	8.16 E–3	1.47	Disease specific survival	GSE17536	212554_at
	177	1.38 E–2	1.59	Disease specific survival	GSE17536	212551_at
	177	3.08 E–2	1.35	Overall survival	GSE17536	212554_at
	145	1.92 E–2	1.73	Relapse free survival	GSE17536	212554_at
	226	3.21 E–3	1.74	Disease free survival	GSE14333	212554_at
	55	3.61 E–2	1.82	Overall survival	GSE17537	212554_at
	55	3.86 E–2	1.98	Disease free survival	GSE17537	212554_at
	49	4.64 E–2	1.98	Disease specific survival	GSE17537	212554_at
**lung**						
	104	1.46 E–2	0.47	Overall survival	Jacob-00182-MSK	212554_at
	104	6.15 E–3	0.16	Overall survival	Jacob-00182-MSK	212554_at
	117	3.69 E–2	1.43	Overall survival	GSE13213	A23P421664
	204	4.64 E–4	0.4	Relapse free survival	GSE31210	212554_at
	204	1.39 E–2	0.55	Relapse free survival	GSE31210	212551_at
**Ovarian**						
	278	1.21E–2	1.38	Overall survival	GSE9891	212554_at
**Prostate**						
	281	4.58E–2	0.82	Overall survival	GSE16560	DAP4_2808
**Skin**						
	38	2.15E–2	1.90	Overall survival	GSE19234	212554_at
	38	3.32E–2	1.66	Overall survival	GSE19234	212554_at

### Protein components of nodes across the CAP

**Figure 9:**
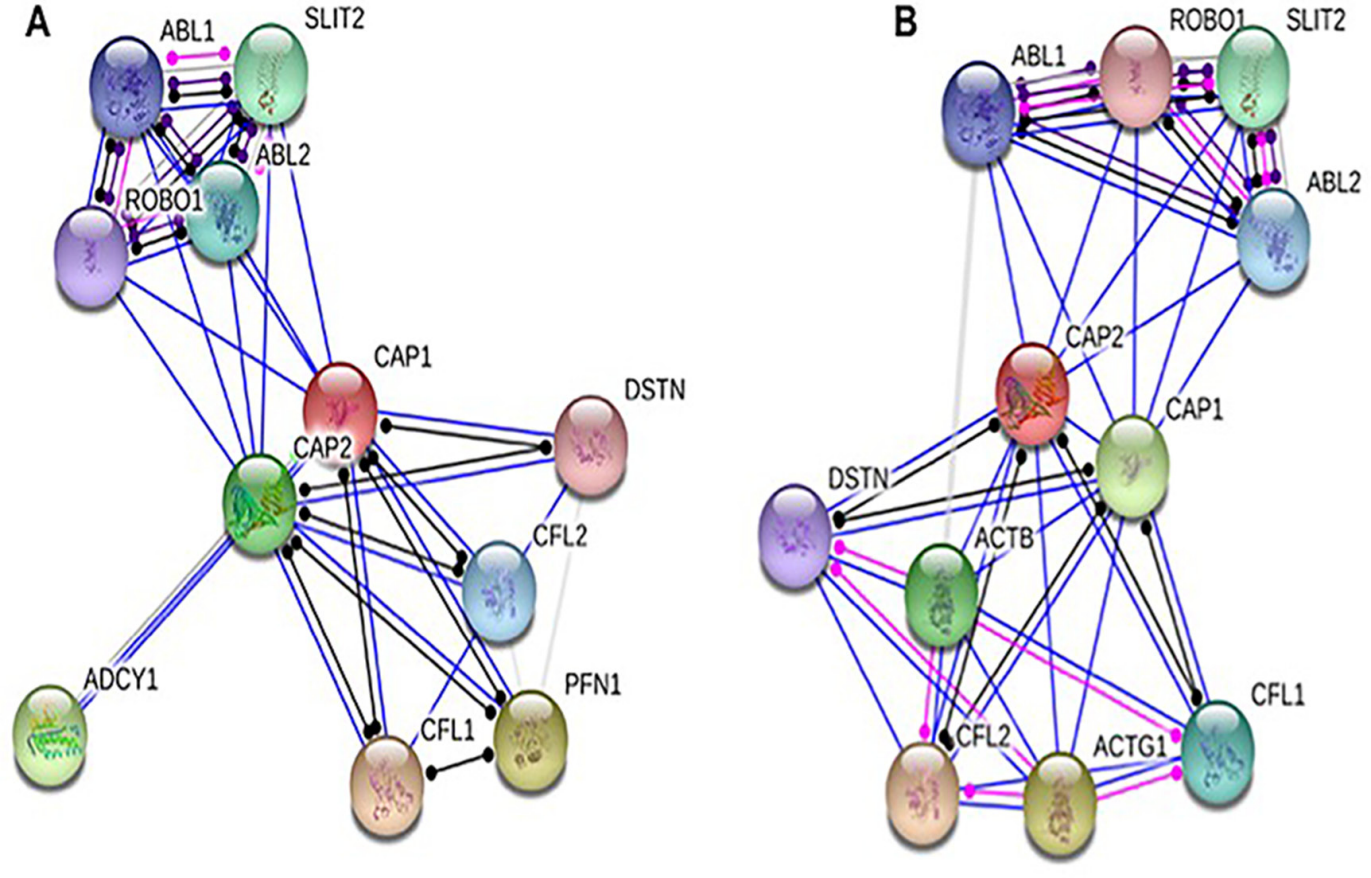
Identification of known and predicted structural proteins essential for CAP function Interacting nodes are displayed in colored circles using String, v10.0. Predicted functional partners of CAP1 (**A**) and CAP2 (**B**) are shown based upon peer reviewed published data and curated database entries. [STRING v.10 (http://string-db.org).

We selected the functional protein partners of CAP based on previous publications and curated databases. The ten predicted proteins of CAP1 (with the corresponding gene names) included: cofilin2 (*CFL2*), CAP2, slit homolog 2 (S*LIT2*), cofilin1 (*CFL1*), v-abl (*ABL2*), c-abl (*ABL1*), destrin (*DSTN*), ROBO1, profilin 1 (*PFN1*), and adenylate cyclase 1 brain (*ADCY1*) (Figure 9A). The ten predicted proteins of CAP2 (with the corresponding gene names) included: cofilin2 (*CFL2*), actin gamma1 (*ACTG1*), CAP1, actin beta (*ACTB*), slit homolog 2 (*SLIT2*), cofilin1 (*CFL1*), v-abl (*ABL2*), c-abl (*ABL1*), destrin (*DSTN*), and ROBO1 (Figure 9B).

CAP homologs are comprised of three conserved structural domains, the N-terminal domain, the C-terminal domain, and a proline-rich central domain [61, 62]. All the three domains contribute to actin filament turnover through interactions with cofilin, and G- and F-actin [62]. In summary, CAP is a key actin-regulating protein that controls actin dynamics through multiple mechanisms including the cofilin-mediated depolymerization cycle [23]. Hence, we choose CAP2, CFL1, CFL2, and DSTN for further analysis of CAP1; CAP1, CFL1, CFL2, DSTN, ACTB, ACTG1 for further analysis of CAP2.

### Unbiased cross cancer subtypes correlations by cBioPortal data

**Figure 10:**
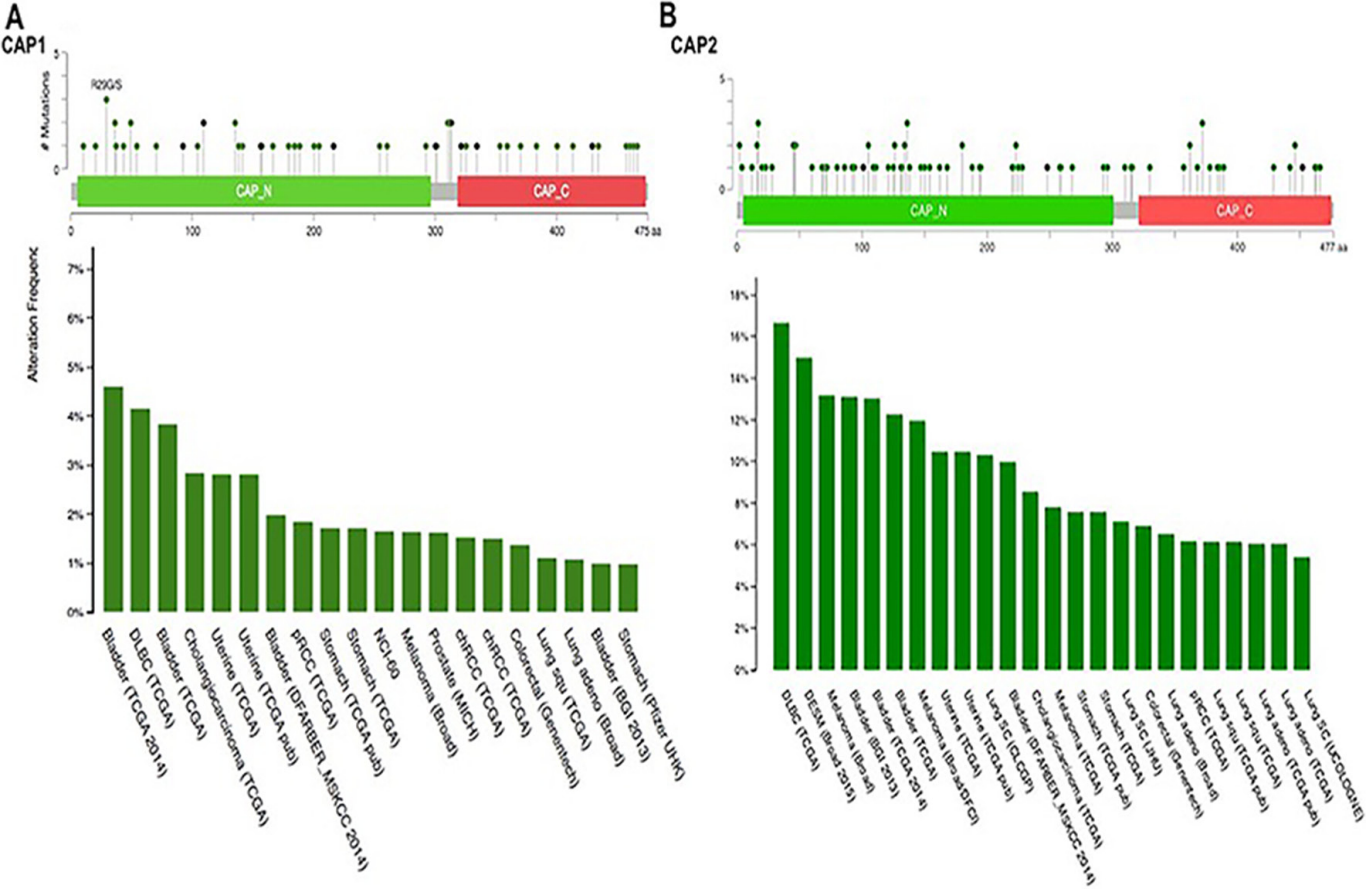
Mutation diagram of CAP in different cancer types across protein domains CAP1 mutation frequencies are the highest in Bladder cancer. The 1 hot spots (R29CVS) represent the common founder mutations in CAP1 N-termial (**A**). CAP2 mutation frequencies are the highest in Myeloma cancer. CAP2 mutation occur in the N domain (**B**).

**Figure 11:**
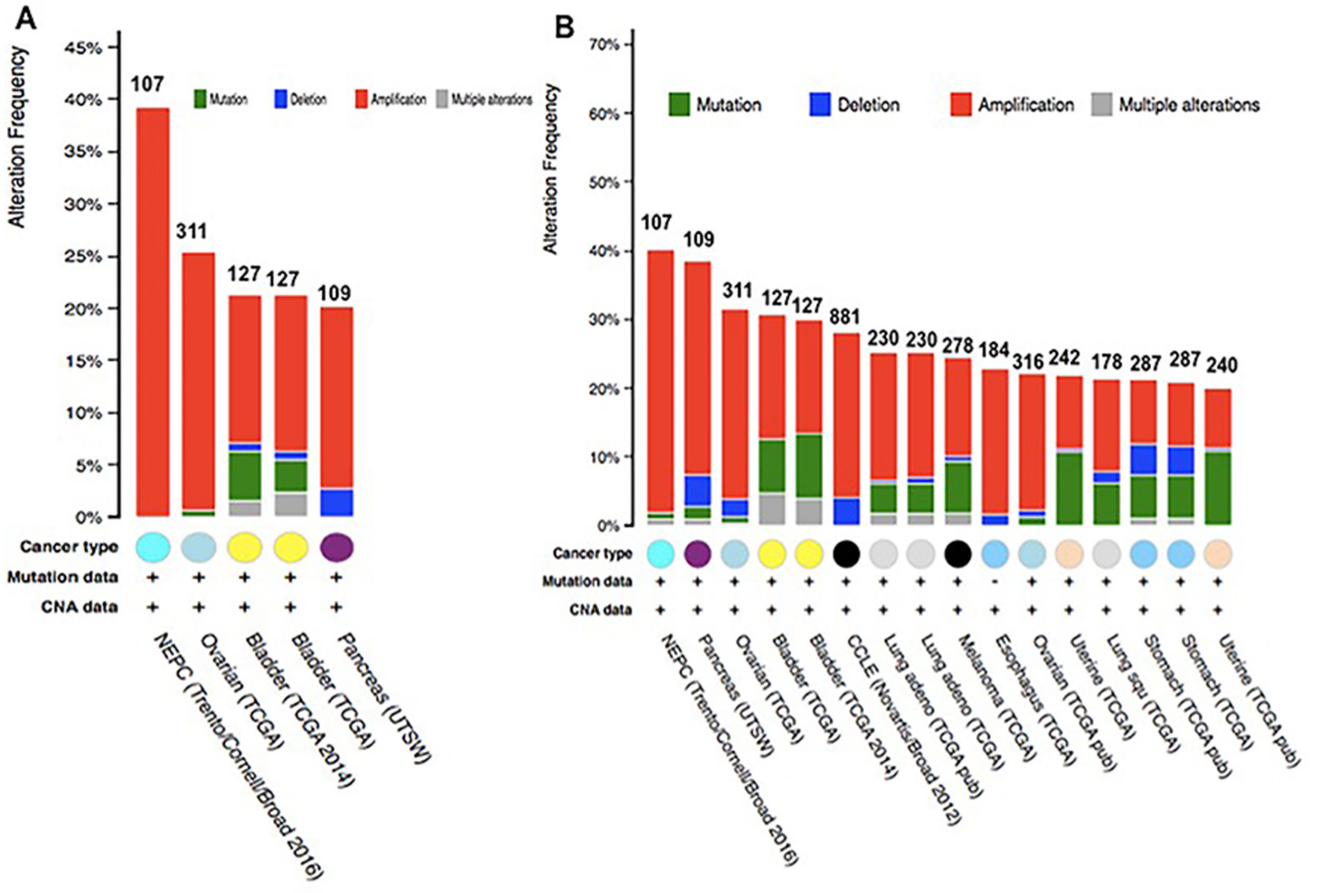
Copy number alteration of CAP genes and cancer subtypes (**A**) the alteration frequency of a five-gene signature (CAP2, CAP1, DSTN, CFL1 CFL2) was determined using the cBioPortal (http://www.cbioportal.org). (**B**) the alteration frequency of a seven-gene signature (CAP2, CAP1, DSTN, CFL1 CFL2, ACTB, ACTG1) was determined using the cBioPortal (http://www.cbioportal.org). Only cancer types containing > 100 samples and an alteration frequency of >20% are shown. The alteration frequency included deletions (blue), amplification (red), multiple alterations (grey) or mutation (green). The total number of samples for each cancer type are indicated by the numbers at the top of each column.

Analyzing the five gene (CAP1) of mutations and CNAs by the cBioPortal tool with 91 different cancer studies. The results analyzed 5 different cancer studies representing 681 samples that contained >20% alteration frequency and at least 100 samples in the dataset. The particular interest constituted the predominant pattern of amplification occurring in prostate cancer. CAP1 mutation mainly occurred in bladder cancer and existed in a hotspot in the CAP N domain (Figure 10A). Minor changes in the deletion or multiple alterations were observed in the results. The ratio of alteration ranged over 39.3–20.2% with the dominance hierarchy (highest to lowest) as prostate, ovarian, bladder, and pancreatic cancer (Figure 11A, Table 6).

**Table 6:** The alteration frequency of a five-gene signature (CAP2, CAP1, DSTN, CFL1 CFL2) in cancers

Cancer	Data source	*N*	Frequency (%)	Amplification (%N)	Deletion (%N)	Mutation (%N)	Multiple alterations (%N)
NEPC	Trento/Cornell/Broad 2016	107	39.3%	39.3% (42)			
Ovarian	TCGA	311	26%	25.42% (79)		0.6% (2)	
Bladder	TCGA 2014	127	21.3%	14.2% (18)	1.6% (2)	4.7% (6)	0.8% (1)
Bladder	TCGA	127	29.9%	14.2% (18)	0.8% (1)	3.1% (4)	2.4% (3)
Pancrease	UTSW	109	20.2%	17.3% (19)	2.8% (3)		

**Figure 12:**
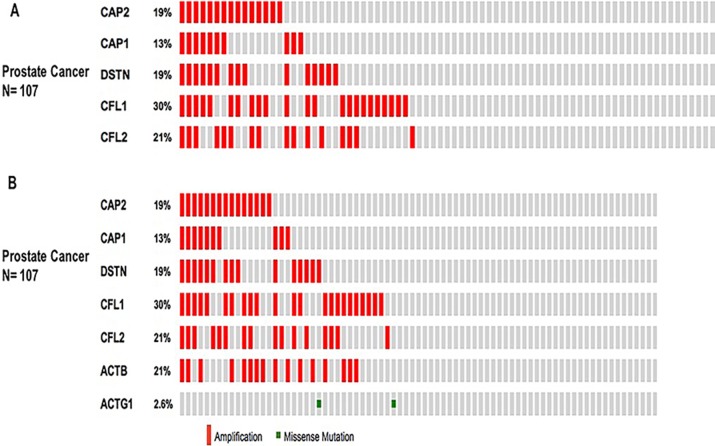
Prostate cancer types frequently amplify CAP We used the Oncoprint feature of the cBioPortal (http://www.cbioportal.org) to determine the copy number alteration frequency of each individual gene in CAP within selected cancer subtypes. The percentages of alterations in *CAP2*, *CAP1*, *CFL1*, *CFL2*, and *DSTN* genes in the prostate cancer (**A**). The percentages of alterations in *CAP2*, *CAP1*, *CFL1*, *CFL2*, *DSTN*, *ACTB*, and *ACTG1* genes among prostate cancer (**B**). Grey bars along a vertical line represent the same sample interrogated for amplification (red), deep deletion (blue), missense mutation (green), truncating mutation (black) or in-frame mutation (brown).

Further, we used the OncoPrint from a query for alterations in *CAP2*, *CAP1*, *CFL1*, *CFL2*, and *DSTN* genes. The percentages of alterations in these genes among prostate cancer varied from 13-30% for individual genes (*CAP2*, 19%; *CAP1*, 13%; *CFL1*, 30%; *CFL2*, 21%; *DSTN*, 19%) (Figure 12A, Table 7); the *CFL1* gene was amplified predominantly in the prostate cancer type.

**Table 7:** The percentages of alterations in CAP2, CAP1, CFL1, CFL2 and DSTN genes

Cancer	CAP1	CAP2	CFL1	CFL2	DSTN
NEPC	13%	19%	30%	21%	19%
Ovarian	11%	12%	2.9%	1.3%	2.9%
Bladder	11%	9%	2.4%	1.6%	0.8%
Bladder	10%	9%	1.6%	1.6%	0.8%
Pancrease	2.8%	4%	10%	5%	4%

The data showed 16 studies representing 4134 samples that contained >20% alteration frequency and at least 100 samples of seven-gene (CAP2) query in the cBioPortal database. A thorough inspection displayed that this result represented approximately ten different cancer types. The predominant pattern of amplification occurring in prostate cancer was of particular interest. The evidence of CAP2 mutation was most predominant in uterine cancer. Also, minor changes in deletion or multiple alterations were observed in the results. Based on the context, it is presumed, that the Cap2 mutation occur in the N domain in the current sentence. (Figure 10B). The frequency of alteration ranged over 40.2–20% with the rank order (highest to lowest) as prostate, pancreatic, ovarian, bladder, and lung cancer followed by melanoma, oesophagus, uterine, and stomach cancer (Figure 11B, Table 8).

**Table 8:** The alteration frequency of a seven-gene signature (CAP2, CAP1, DSTN, CFL1 CFL2, ACTB, ACTG1) in cancers

Cancer	Data source	*N*	Frequency (%)	Amplification (%N)	Deletion (%N)	Mutation (%N)	Multiple alterations (%N)
NEPC	Trento/Cornell/Broad 2016	107	40.2%	38.3% (41)		0.9% (1)	0.9% (1)
Pancrease	UTSW	109	38.5%	31.2% (34)	4.6% (5)	1.8% (2)	0.9% (1)
Ovarian	TCGA	311	32.5%	28.3% (88)	2.9% (9)	1% (3)	0.3% (1)
Bladder	TCGA 2014	127	29.9%	16.5% (21)		9.4% (12)	3.9% (5)
Bladder	TCGA 127	127	29.9%	17.3% (22)		7.9% (10)	4.7% (6)
CCLE	Novartis/ Broad 2012	881	24.1% (212)			4.1% (36)	
Lung adeno	TCGA	230	29.9% (44)	17.3% (10)		7.9% (4)	4.7% (1)
Melanoma	TCGA	287	25.7%	19.1% (44)	4.3% (23)	0.4% (2)	1.7% (4)
Lung adeno	TCGA pub	230	25.4%	15.3%	0.7% ()	8% ()	1.4% ()
Esophagus	TCGA	184	25.2%	20.7% (38)	1.6% (3)	2.7% (5)	
Ovarian	TCGA pub	316	25%	19.9% (63)	0.9% (3)	1.3% (4)	
Lung Squ	TCGA	177	22%	14.1% (25)	1.7% (3)	5.6% (10)	0.6% (1)
Uterine	TCGA	242	21.9%	10.7% (26)	0.4 (1)	10.7 (26)	
Stomach	TCGA pub	287	21.6%	9.1% (26)	4.5% (13)	6.6% (19)	1.4% (4)
Uterine	TCGA pub	240	20%	8.8% (21)	0.4% (1)	10.8% (26)	

**Figure 13:**
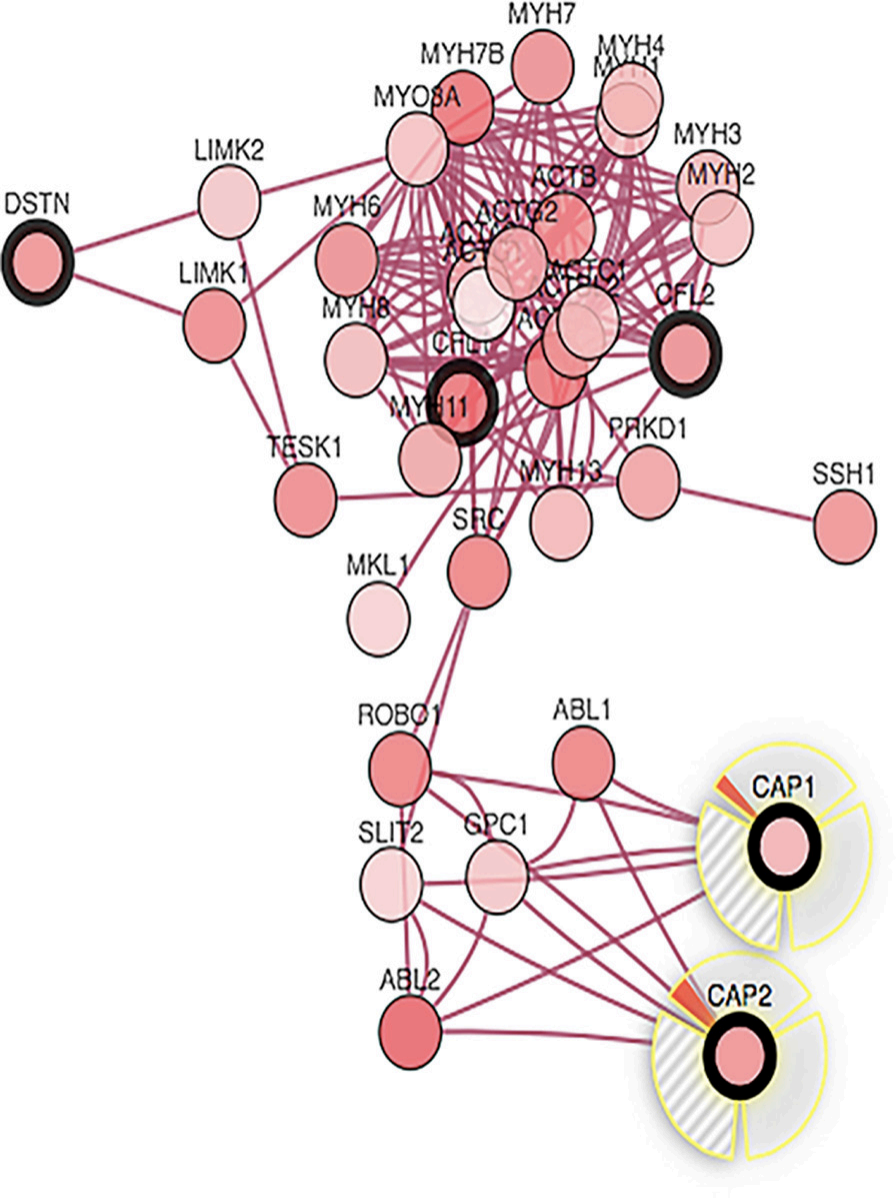
The interactions between CAP1 and CAP2 alteration (cBio Cancer Genomics Portal) Network view of the CAP1/CAP2 neighborhood in prostate cancer. CAP1 and CAP2 are seed genes (indicated with thick border), and all other genes are automatically identified as altered in prostate cancer. Darker red indicates increased frequency of alteration (defined by mutation, copy number amplification, or homozygous deletion) in prostate cancer.

We also applied the OncoPrint database to explore the specific alterations in each gene. For example, percentages of alterations in *CAP2, CAP1*, *CFL1*, *CFL2*, *DSTN*, *ACTB*, and *ACTG1* genes among prostate cancer varied from 2.6–30% in individual genes (*CAP2*, 19%; *CAP1*, 13%; *CFL1*, 30%; *CFL2*, 21%; *DSTN*, 19%; *ACTG1*, 2.6%; *ACTB*, 21%;) (Figure 12B, Table 9); the *CFL1* gene was amplified predominantly in prostate cancer. We also use Tumorscape database to verify the copy number changes in cancers. Figure 13 shows the detail of CAP genomic regions that are either significantly amplified or deleted in specific cancer.

**Table 9:** The percentages of alterations in CAP2, CAP1, CFL1, CFL2, DSTN, ACTB, and ACTG1 genes

Cancer	ACTB	ACTG1	CAP1	CAP2	CFL1	CFL2	DSTN
NEPC	21%	2.6%	13%	19%	30%	21%	19%
Pancrease	17%	10%	2.8%	4%	10%	5%	4%
Ovarian	4%	8%	11%	12%	2.9%	1.3%	2.9%
Bladder	7%	6%	11%	9%	2.4%	1.6%	0.8%
Bladder	8%	6%	10%	9%	1.6%	1.6%	0.8%
CCLE	9%	6%	4%	4%	4%	5%	3%
Lung adeno	8%	4%	1.7%	3%	0.4%	13%	1.7%
Melanoma	7%	7%	1.4%	10%	4%	0.7%	1%
Lung adeno	8%	4%	1.7%	3%	0%	12%	1.7%
Esophagus	9%	3%	5%	2.2%	2.7%	2.7%	1.6%
Ovarian	1.3%	4%	10%	7%	0.3%	1.3%	1.3%
Lung Squ	2.3%	6%	3%	4%	1.1%	3%	4%
Uterine	2.9%	6%	6%	7%	2.1%	2.9%	3%
Stomach	8%	1.7%	3%	5%	1.4%	3%	3%
Uterine	3%	6%	5%	7%	1.3%	3%	2.9%

In order to find whether the identified correlation is significant for each gene pair, the portal performs a Fisher's exact test. The mutual exclusivity analysis showed that the events in the selected genes co-occur, and the pattern was statistically significant between CAP2 and CAP1, CFL1, CFL2, ACTB, DSTN, but not between CAP2 and ACTG1, the main reason for lower correlation between CAP2 and ACTG1 seems to be the lower mutation rate in ACTG1, as showed in Figure 12B, the percentage of alteration of ACTG1 gene is only 2.6% that is quite small compared with other genes (∼20%) in 107 samples. Functional plotting of the corresponding mRNA level associated with the genetic status of CAP1and CAP2 revealed that deletion of these two CAPs was associated with increased mRNA expression (Supplementary Figure 9).

The cBioPortal analysis program identified 12 types of human cancer with significant CNAs in the chosen genes’ signature (CAP1, CAP2, CFL1, CFL2, DSTN). The CAP signature was created such as to represent the structures and functions of CAP. The CNAs of specific structural components of the CAP in tumors may be potential targets to prevent metastatic spread.

Furthermore, we analyzed the interactions between CAP1 and CAP2 alteration via computation to reveal a moderate strength of direct interaction (Figure 13).

**Figure 14:**
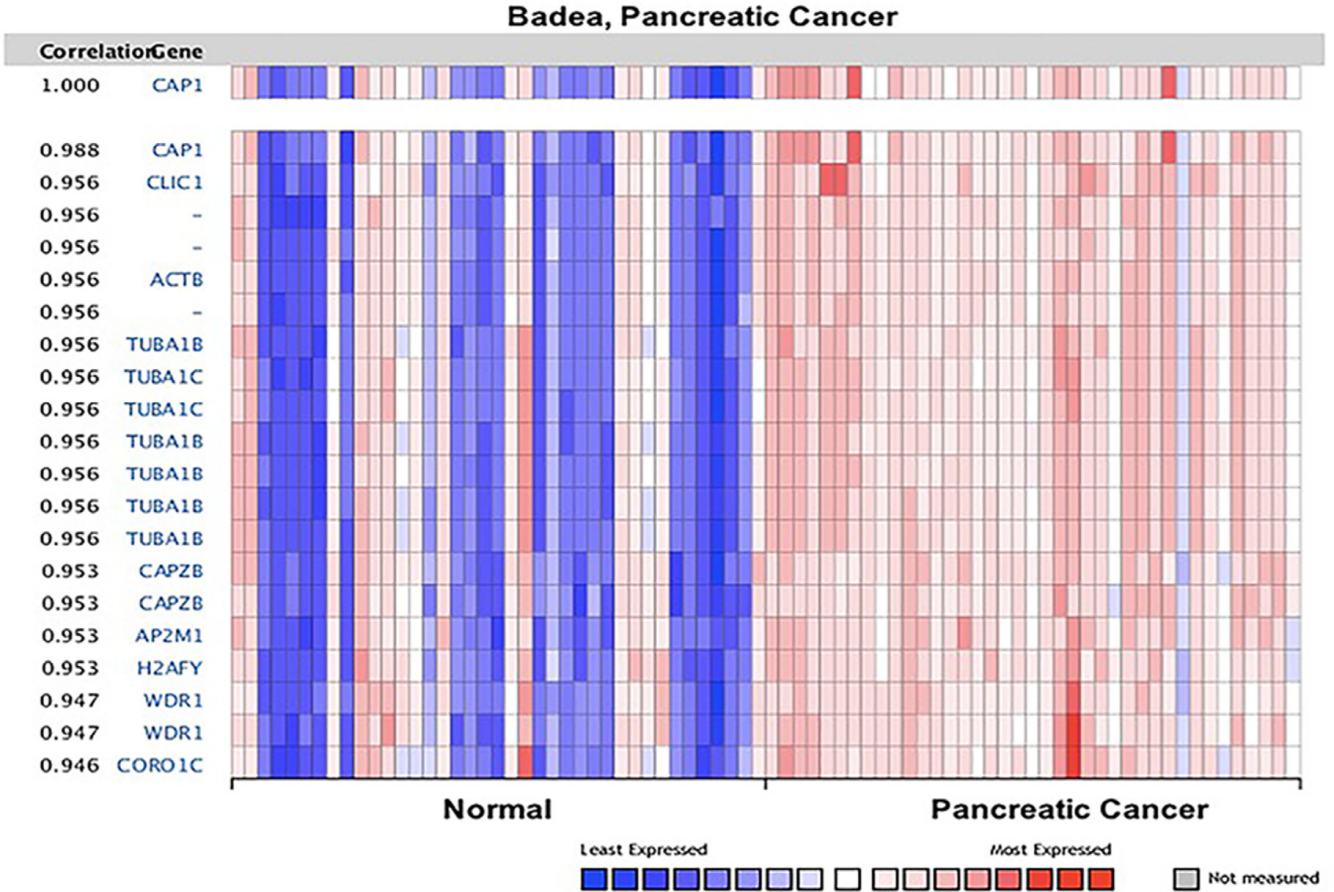
CAP1 genes in Pancreatic cancer CAP1 is coexpressed with the indicated genes across a panel of 39 pancreatic and 39 normal samples. Bar length represented the significance and negative logarithm of enrichment *p*-value.

**Figure 15:**
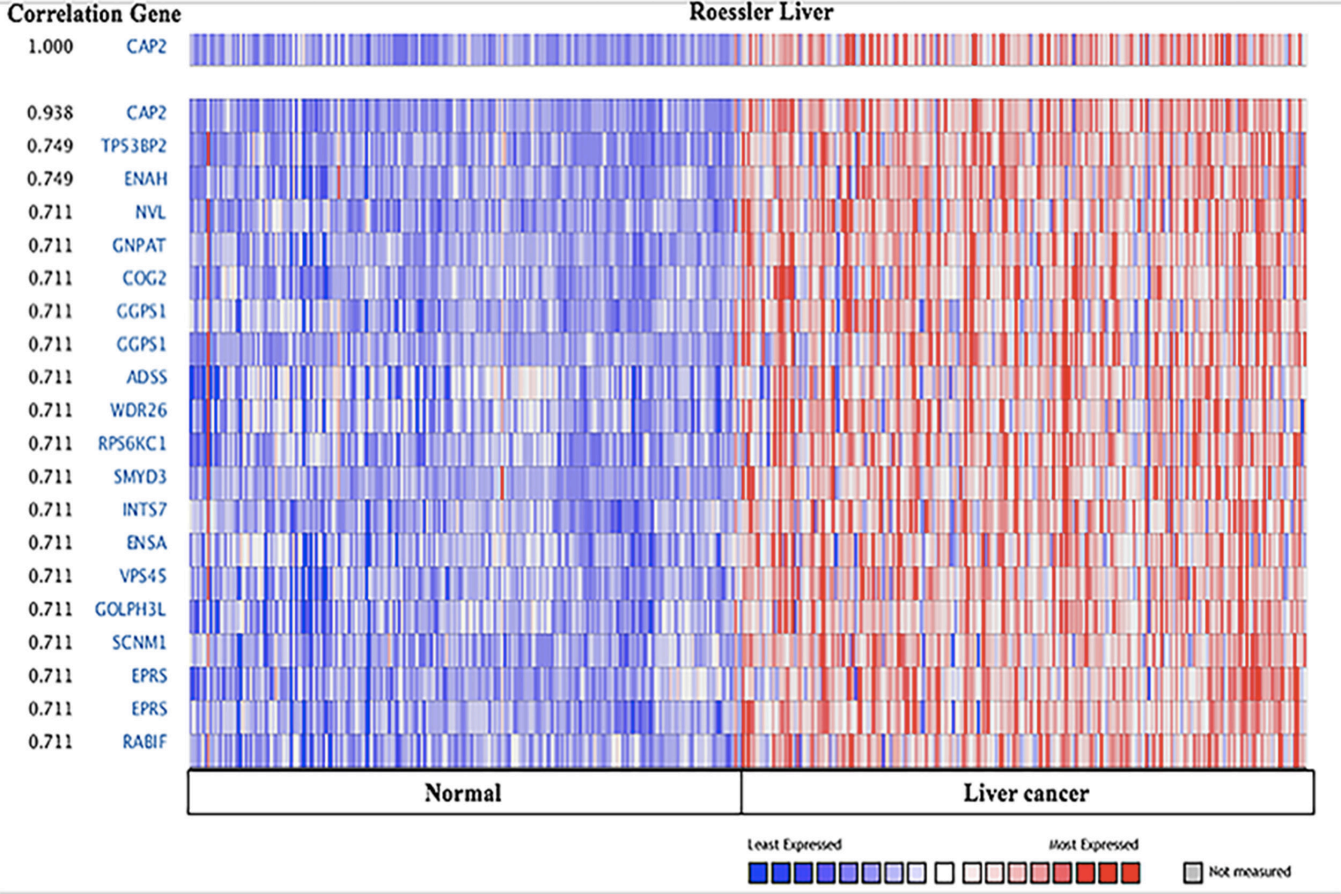
CAP2genes in Liver cancer CAP1 is coexpressed with the indicated genes across a panel of 225 Liver and 220 normal samples. Bar length represented the significance and negative logarithm of enrichment *p*-value.

The co-expression of CAP were analyzed by Oncomine (Figures 14, 15, Supplementary Figures 10, 11). The co-expression profile of CAP1 was identified with a big cluster of 127 genes across 39 pancreatic carcinomas and 39 normal pancreatic tissues (Figure 14), as well as 52 genes across 32 head-neck and 26 normal samples (Supplementary Figure 10). CAP1 was co-expressed with Tubulin alpha-1B chain (*TUBA1B*) in both pancreatic and head-neck cancer. TUBA1B expression has been reported to be unregulated in liver tumor tissues, and an increased TUBA1B expression was associated with poor overall survival and resistance to paclitaxel in liver cancer patients [63].

In addition, we explored the co-expression profiles for CAP2 with 20 genes across 225 liver carcinomas and 220 normal liver tissues (Figure 15), and 20 genes across 157 brain and 23 normal samples. Additionally, 17 genes across 89 prostate cancers and 23 normal prostate tissues were also discovered (Supplementary Figure 11). Interestingly, CAP2 was coexpressed with tumor protein p53 binding protein 2 (*TP53BP2*) and ENA/VASP expressed in liver cancer. TP53BP2 was associated with several types of cancers. [63]. TP53BP2 is a key regulator of epithelial plasticity that connects cell polarity to suppress tumor metastasis [64]. Overexpression of TP53BP2 promoted the proliferation in breast cancer cells [65, 66]. On the other hand, ENA/VASP proteins are actin-associated proteins implicated in series of processes rely on cytoskeleton remodeling and cell polarities, such as axon guidance and lamellipodia and filopodia dynamics in migrating cells [67–69]. The GO analyses demonstrated the potential pathway of CAP1 and CAP2 by ICGC (Supplementary Figure 12) [70, 71]. CAP is involved in actin binding, cell morphogenesis, and cell migration. The precise underlying mechanism through which CAP1 and CAP2 modulate cancer progression need to be further studied.

## DISCUSSION

CAP has been proved to prompt tumor development in cancers [7–10]. Despite scores of CAPs identified so far, little is known whether they can serve as markers for cancer diagnosis/prognosis. In order to establish the compelling evidence, in the present study, we conducted the data depend on plenty of genes expression with clearly defined parameters between cancer and normal tissues. In the Oncomine analysis, CAP1 was found to be unregulated in various cancer types (Table 1, Figure 1), but deregulated in leukemia and breast cancer. CAP2 was deregulated in various cancer types (Table 3, Figure 1), but unregulated in liver, gastric, kidney, and breast cancer.

To further explore the OS between CAP and various types of cancer, the correlations between CAP gene and survival rates was evaluated using the Kaplan-Meier Plotter and PrognoScan. Overall, high levels of CAP1 gene expression result in low survival in breast and ovarian cancers (Table 2). However, the results between OS and lung cancer is not clear. Our previous study confirmed the role of CAP1 in lung cancer demonstrated that the expression of CAP1 was significantly higher in NSCLC tissues as compared to the corresponding normal lung tissues. Moreover, CAP2 gene expression led to increased survival.

Somatically acquired genetic, epigenetic, transcriptomic, and proteomic alterations are the main four causes of cancer cells [72]. These alterations occur in specific genomic regions, which could show their potential suppressive or oncogenic roles [73]. Thus, the cBioPortal analysis to identify human cancers discovered significant CNA in the chosen CAP-gene signature. The present study focused on a specific group of CAP from HGNC to examine the prostate cancer dataset (provisional) on the cBioPortal. The cBioPortal analysis found that the frequency of 40.2–20% with the rank order mainly in prostate cancer with CAP1 and CAP2 alteration ranged from 38.3–19.8%. The CAP mutations occurs in the N domain of CAP (Figures 11, 12).

The cBioPortal can be used for interactive analysis and visualization of altered networks. The networks consist of pathways and interactions from the Human Protein Reference Database [74], Reactome [75], NCI Pathway Interaction Database [76], and the MSKCC Cancer Cell Map [77]. Figure 13 showed that the Network view of the CAP1/CAP2 neighborhood in prostate cancer, those results its better to understand the molecular mechanisms of CAP underlying cancer.

The Oncomine™ database displays a potentially significant list of coexpressed genes, which is critical in defining pathways. Co-expression analysis revealed that CAP1 was coexpressed with TUBA1B both in pancreatic and head-neck cancer (Figure 14), as well as with CFL1, CFL2, DSTN, and ROBO1, according to STRING analysis. CAP2 was coexpressed with TP53BP2, ENA/VASP in the liver cancer (Figure 15), and CFL1, CFL2, DSTN, ACTB, ACTG1, and ROBO1. In addition, our previous studies found that CAP1 was overexpressed in NSCLC tissues and correlated with poor clinical outcomes [7]. In order to clarify the molecular mechanisms of CAP1 on the metastasis of NSCLC cells, our previous studies showed that CAP1 was a phosphorylatable protein. Zhou et al. found that homologs of CAP1 had four phosphorylation sites: S36, S307, S309, and T314. CAP1 regulates cancer mechanism through Tandem phosphorylation of S307 and S309 and association with cofilin and actin by GSK3 in HeLa cells [24]. Zhang et al. also found that CAP1 promotes breast cancer cell proliferation and metastasis mediated by ERK [78]. These mechanistic insights may ultimately lead to therapeutic strategies targeting CAP1 or its peripheral cell signals in cancer treatment.

As is known to all, this is the first study indicating the function of CAP members in cancer development. The present study facilitated access and interpretation of multidimensional oncogenic data. The use of the portals contributes to a better understanding of cancer molecular etiology and epidemiology that will ultimately accelerate the translation of genomic knowledge into clinical practice [79]. The current study aimed to show extensive oncogenic databases for the better understanding of the molecular mechanisms. In addition, we show an explicit direction through several web-based oncogenic portals that were established to facilitate researchers from different cancer-associated fields. Descriptions of the specific portals prepared on the basis of their versions from 2016 were summarized in Table 5.

## MATERIALS AND METHODS

### Oncomine database analysis

The expression level of CAP gene in various types of cancers was identified from Oncomine database (https://www.oncomine.org/resource/login.html) [13, 14]. The mRNA expression fold in cancer tissue compared to the normal tissue was obtained as the parameters of *p*-value < 1E-4, fold change > 2, and gene ranking in the top 10% and the analyses were summarized in Tables 1, 3. The co-expression profiles of CAP gene in different types of cancers were identified and presented as the pattern of heat map.

### Kaplan-Meier plotter database analysis

The Kaplan Meier plotter is capable to assess the effect of 54,675 genes on survival using 10,188 cancer samples (4,142 breast, 1,648 ovarian, 2,437 lungs and 1,065 gastric cancer) on the HGU133 Plus 2.0 array. The correlation between CAP expression and survival in breast, gastric, ovarian and lung was analyzed by Kaplan-Meier plotter (http://kmplot.com/analysis/) [15]. The hazard ratio with 95% confidence intervals and log rank *p*-value was also computed.

### Prognoscan database analysis

The correlation between CAP expression and survival in various types of cancers was analyzed by PrognoScan database (http://www.abren.net/PrognoScan/) [16]. The threshold was adjusted to cox *p*-value < 0.05 and the analyses were summarized in Tables 2, 4.

### Identifying the protein components of CAP axis

The STRING analysis tool was used to determine interacting proteins using CAP as the query (http://string-db.org). The adenylate cyclase-associated protein (Homo saplens) was used. Several known partners have been genetically verified and therefore served as the foundation for finding the other protein partners in the axis. Any proteins identified that were not specific to the CAP axis, (e.g., slit homolog 2 [SLIT2]) were excluded from the gene signature [17, 18].

### cBioPortal database analysis

We utilized the ability to conduct an integrative analysis of CAP gene and clinical characteristic using the cBioPortal data, an open access resource at http://www.cbioportal.org/ [19, 20], currently provided access to data from more than 5,000 tumor samples from 105 cancer studies in the TCGA pipeline. The query interface combined with customized data storage enabled us to interactively explore genetic alterations across samples curated from national and international cancer studies and specific genes. The primary search parameters included alterations (amplification, deep deletion, missense mutations), CNA from GISTIC and RNA seq data with the default setting. For the secondary search, we focused on RNA seq data.

### Tumorscape database analysis

Tumorscape [21, 22] was developed at The Broad Institute of MIT and Harvard in Cambridge, MA USA (http://www.broadinstitute.org/tumorscape/). This website was one of the first oncogenomic portals to provide information about cancer copy number changes in a format that was easily accessible to non- bioinformaticians. With this portal, the copy number profiles of over 3,700 cancers (both primary cancers and cell lines) are mapped to the human genome reference sequence and are visualized as heatmap tracks, with the use of the Integrative Genomics Viewer (The Broad Institute). Genomic regions with increased (> 2) and decreased (< 2) copy number are marked, respectively, in red and blue colors, the intensity of which indicates the amplitude of the copy number changes. The tracks that represent all of the analyzed samples are shown next to one another, forming a panel that allows direct comparison and visualization of all of the analyzed samples. In addition, Tumorscape provides tools that allow “cancer-centric” and “gene-centric” data analyses.

### Statistical analysis

The results were performed using GraphPad Prism version 6 (GraphPad Software, La Jolla, CA, USA). Survival curves generated by the cBioPortal and Kaplan-Meier plots. All results are displayed with *P* values from a log-rank test. Similarly, with Oncomine, heat maps. Statistical significance of the data (*P*-values) was provided by the program.

## CONCLUSIONS

In summary, bioinformatics analyses with Kaplan-Meier plotter, PrognoScan, cBioPortal, STRING analysis, Oncomine, Tumorscape, and ICGC Data Portal indicates that CAP is implicated in the cancer progressive. The inhibitor or activator of CAP for cancer treatment is based on different cancer types. Furthermore, by learning the databases of this study, researchers can explore the signaling network of CAP in cancer or other diseases.

## SUPPLEMENTARY MATERIALS FIGURES AND TABLES



## Abbreviations

CAP: adenylate cyclase-associated protein
NSCLC: non-small cell lung cancer
CNA: copy number alteration
ICGC: International Cancer Genome Consortium
CFL1: cofilin1
CFL2: cofilin2
DSTN: destrin; ROBO1
TUBA1B: Tubulin alpha-1B chain
TP53BP2: tumor protein p53 binding protein, 2
ENA/VASP: ACTB, actin, beta
ACTG1: actin, gamma1
v-abl: ABL2
c-abl: ABL1
