# Supplementary material for: Systematic analysis of gene expression alterations and clinical outcomes of adenylate cyclase-associated protein in cancer
Source: Oncotarget. 2017 Mar 10;8(16):27216–39. doi: 10.18632/oncotarget.16111 (PMC5432330; doi:10.18632/oncotarget.16111)
Supplement: Supplementary file 1 [file oncotarget-08-27216-s001.pdf]

Systematic analysis of gene expression alterations and clinical outcomes of adenylate cyclase-associated protein in cancer

Supplementary Materials

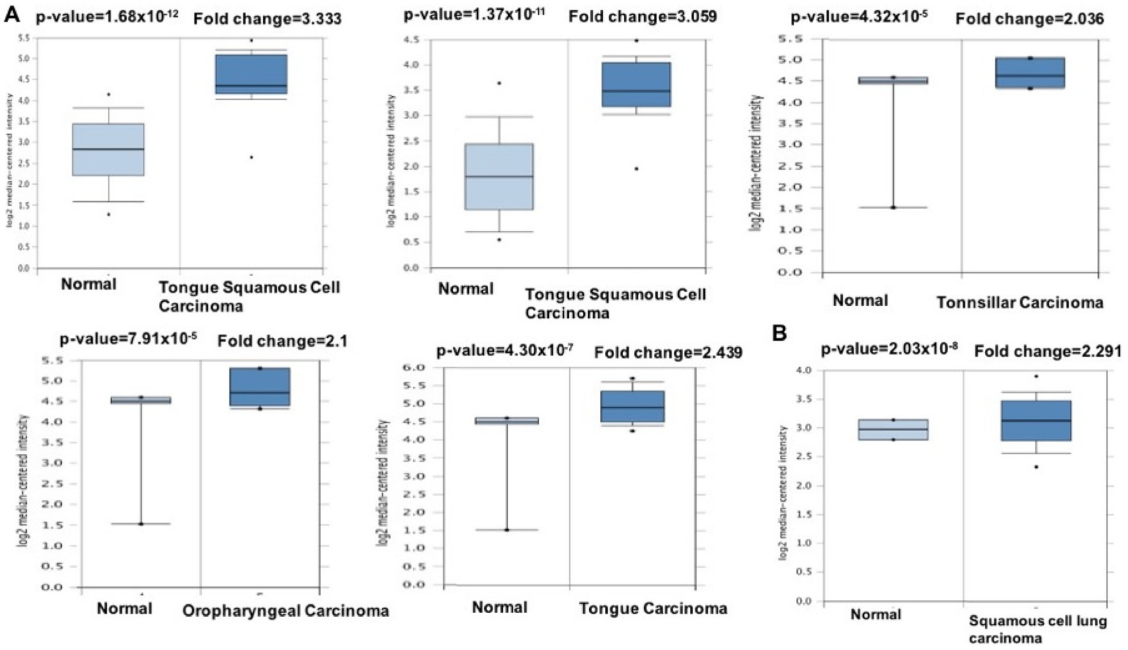

Supplementary Figure 1: CAP1 gene analysis in Head-Neck and lung cancer (Oncomine database). The box plot comparing specific CAP1 expression in normal (left plot) and cancer tissue (right plot) was derived from Oncomine database. The analysis was shown in Head-Neck carcinoma relative to normal Head-Neck (**A**), in lung carcinoma relative to normal lung (**B**).

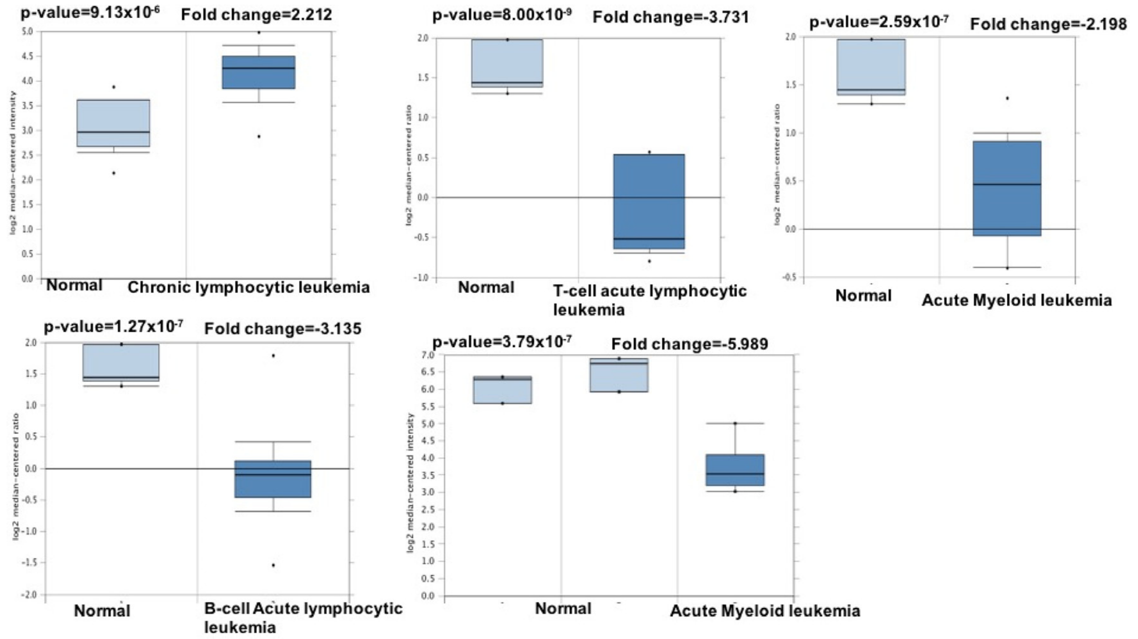

Supplementary Figure 2: CAP1 gene analysis in Leukemia cancer (Oncomine database). The box plot comparing specific CAP1 expression in normal (left plot) and cancer tissue (right plot) was derived from Oncomine database. The analysis was shown in leukemia carcinoma relative to normal tissue.

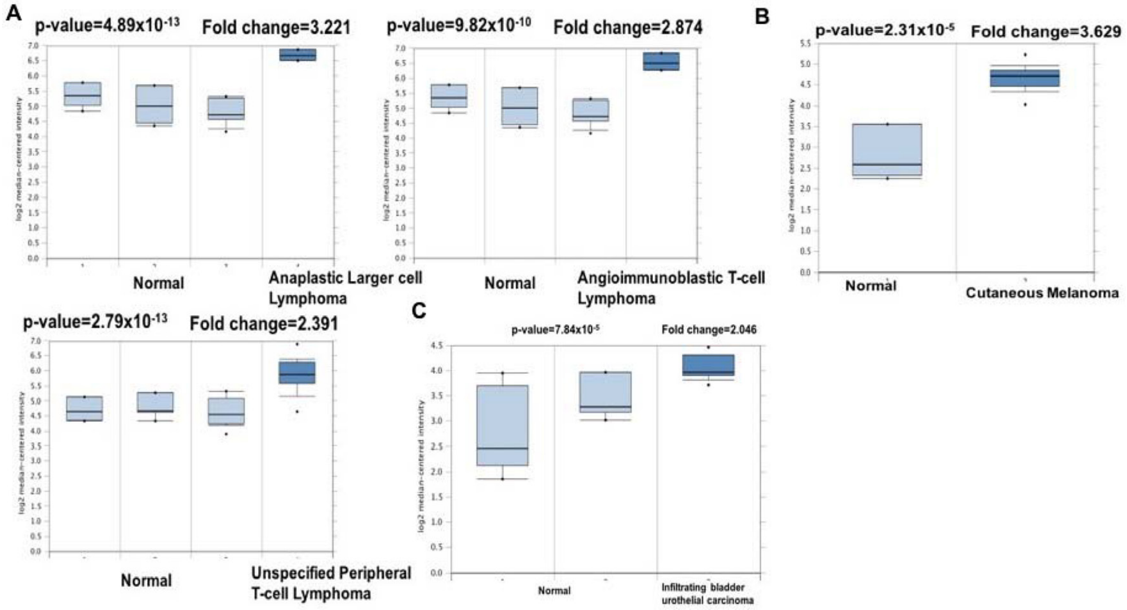

Supplementary Figure 3: CAP1 gene analysis in Lymphoma, Melanoma and Bladder cancer (Oncomine database). The box plot comparing specific CAP1 expression in normal (left plot) and cancer tissue (right plot) was derived from Oncomine database. The analysis was shown in Lymphoma carcinoma relative to normal tissue (**A**), in Melanoma relative to normal tissue (**B**). in Bladder cancer relative to normal bladder (**C**).

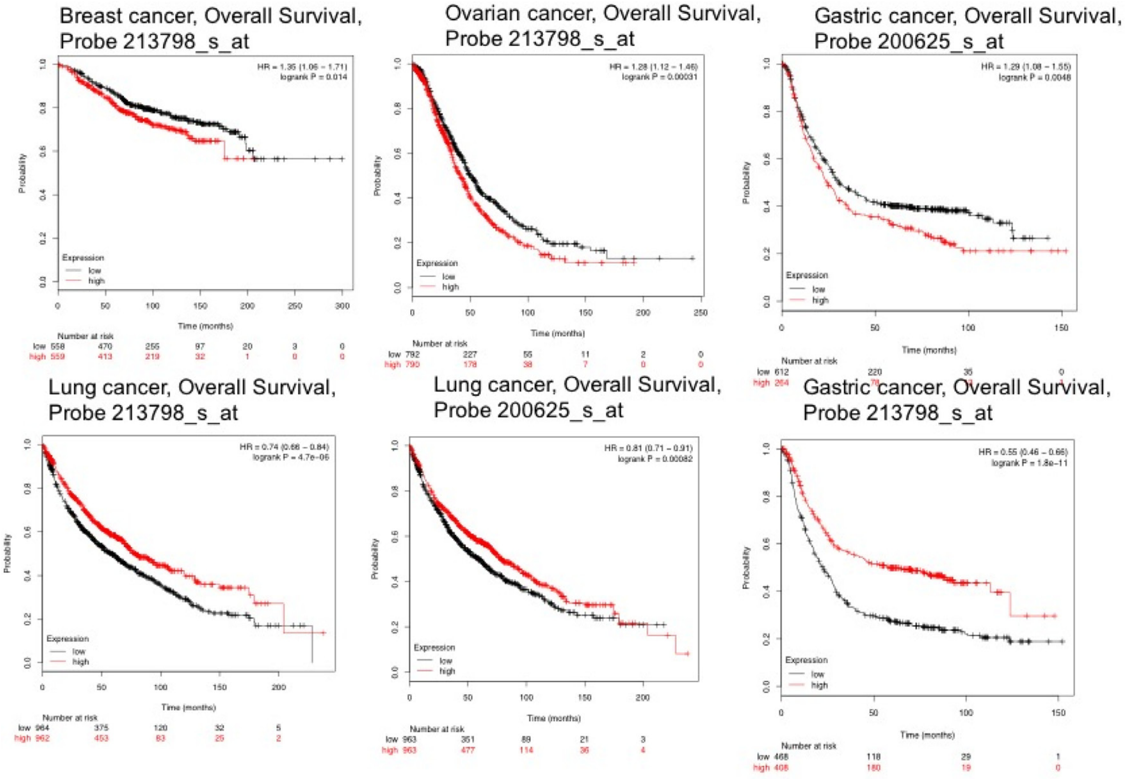

Supplementary Figure 4: CAP1 genes in Breast, Ovarian, Gastric and Lung cancer (Kaplan-Meier Plotter). The survival curve comparing the patient with high (red) and low (black) expression in breast, ovarian, gastric and lung cancer was plotted from Kaplan-Meier plotter database.

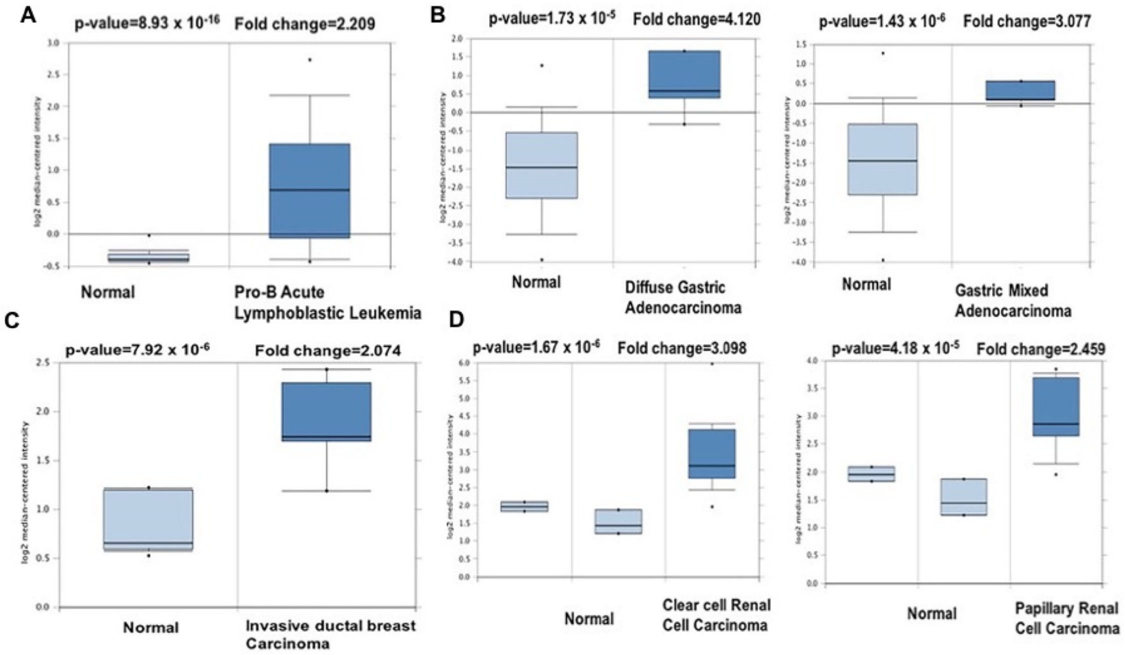

Supplementary Figure 5: CAP2 gene analysis in Leukemia, Gastric, Breast and Renal cancer (Oncomine database). The box plot comparing specific CAP2 expression in normal (left plot) and cancer tissue (right plot) was derived from Oncomine database. The analysis was shown in Leukemia relative to normal (**A**), in gastric carcinoma relative to normal gastric (**B**), in in breast carcinoma relative to normal breast (**C**), in renal carcinoma relative to normal renal (**D**).

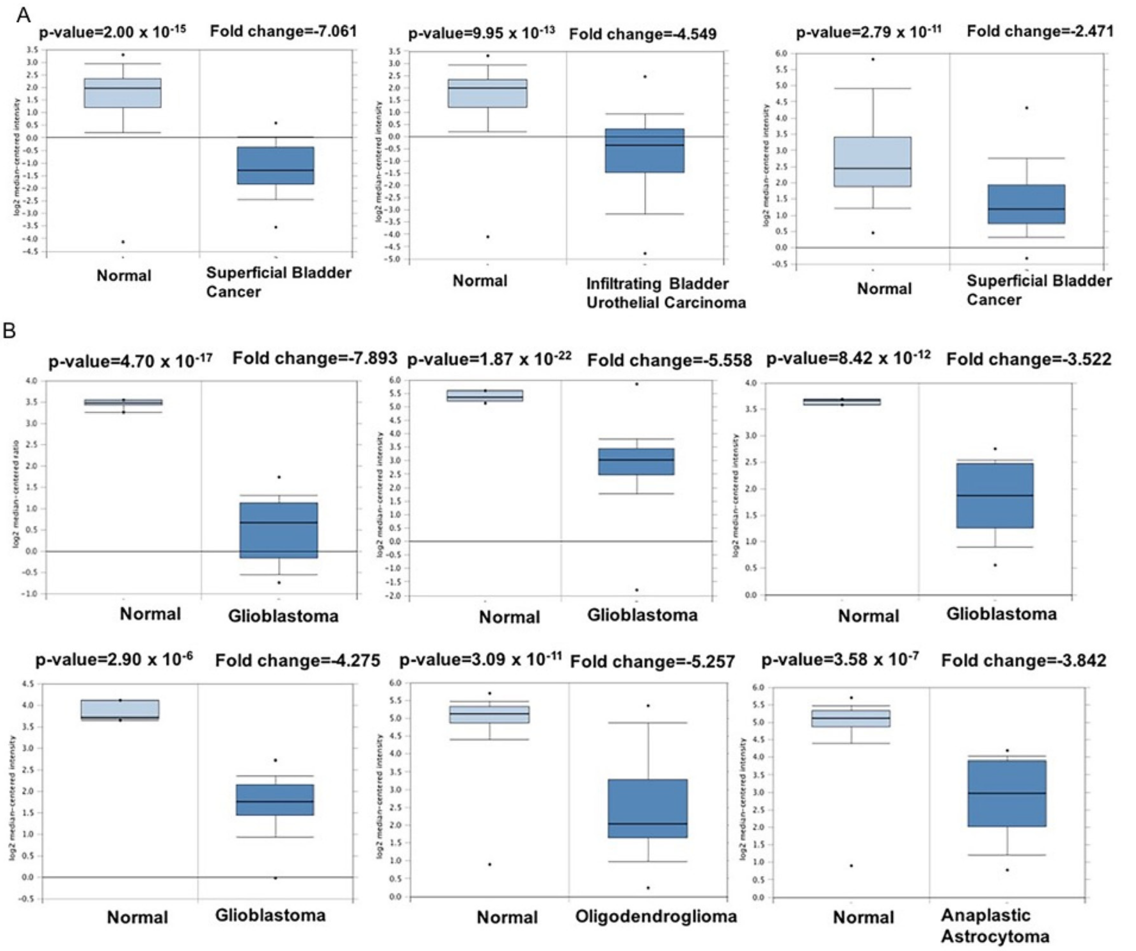

Supplementary Figure 6: CAP2 gene analysis in Bladder and Renal cancer (Oncomine database). The box plot comparing specific CAP2 expression in normal (left plot) and cancer tissue (right plot) was derived from Oncomine database. The analysis was shown in Bladder relative to normal bladder (**A**), in Glioblastoma relative to normal tissue (**B**).

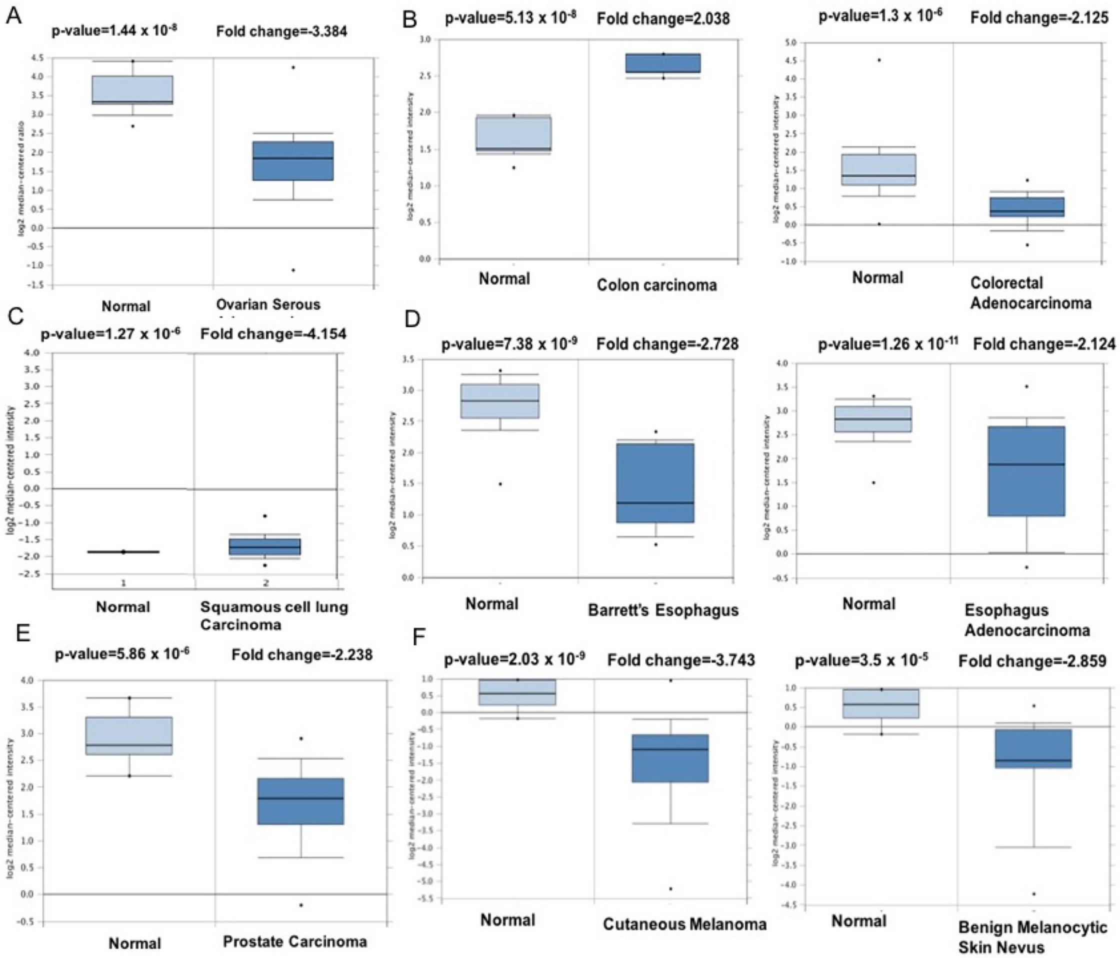

Supplementary Figure 7: CAP2 gene analysis in Ovarian, Colon, Lung, Esophagus, Prostate and Melanoma cancer (Oncomine database). The box plot comparing specific CAP2 expression in normal (left plot) and cancer tissue (right plot) was derived from Oncomine database. The analysis was shown in Ovarian relative to normal ovarian (**A**), in Colorectal relative to normal tissue (**B**), in Lung cancer relative to normal ovarian (**C**), in Esophagus relative to normal tissue (**D**), in Prostate cancer relative to normal prostate (**E**), in Melanoma relative to normal tissue (**F**).

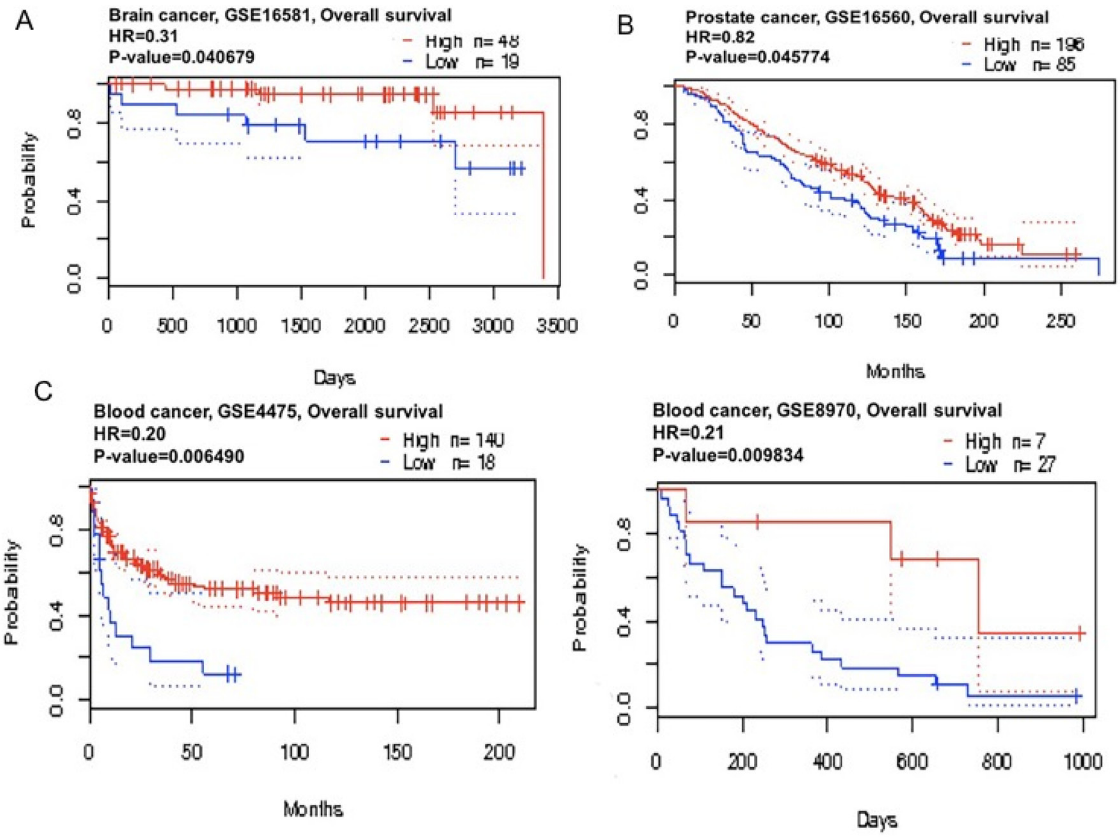

Supplementary Figure 8: CAP2 genes in Brain, Prostate and Blood cancer (PrognoScan database). The survival curve comparing the patient with high (red) and low (black) expression in brain, prostate and blood cancer were plotted from PrognoScan database as the threshold of cox *p*-value < 0.05.

A

Gene A	Gene B	p-Value	Log Odds Ratio	Association	
CAP2	CAP1	<0.001	2.686	Tendency towards co-occurrence	Significant
CAP2	DSTN	<0.001	2.130	Tendency towards co-occurrence	Significant
CAP2	CFL1	<0.001	2.251	Tendency towards co-occurrence	Significant
CAP1	DSTN	<0.001	2.805	Tendency towards co-occurrence	Significant
CAP1	CFL2	<0.001	2.713	Tendency towards co-occurrence	Significant
DSTN	CFL1	<0.001	1.901	Tendency towards co-occurrence	Significant
DSTN	CFL2	<0.001	2.159	Tendency towards co-occurrence	Significant
CFL1	CFL2	<0.001	2.057	Tendency towards co-occurrence	Significant
CAP2	CFL2	0.001	2.031	Tendency towards co-occurrence	Significant
CAP1	CFL1	0.013	1.754	Tendency towards co-occurrence	Significant

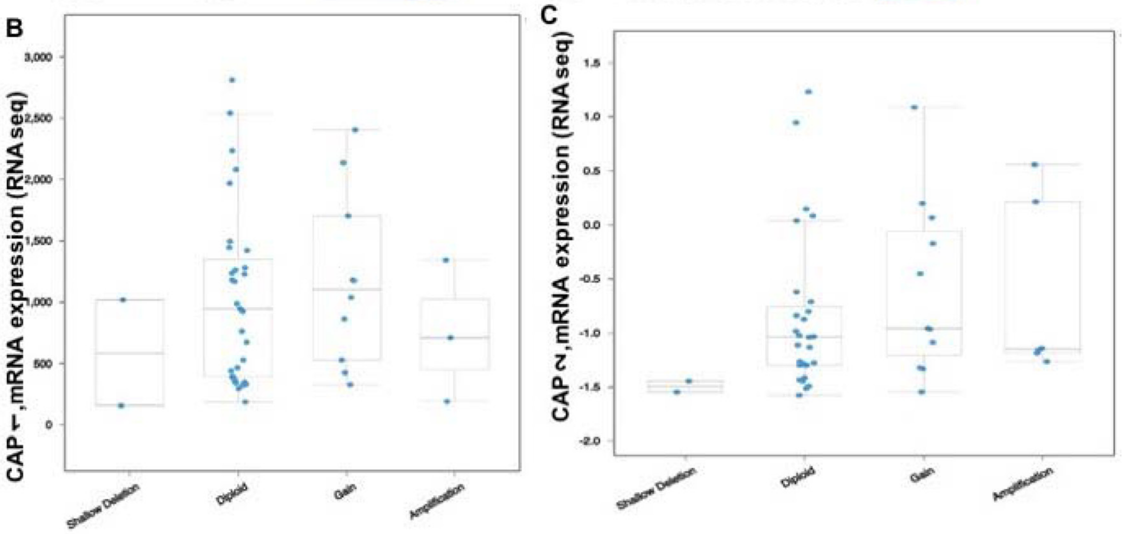

Supplementary Figure 9: The example shows that genes that alter CAP1, CAP2 in Prostate have a tendency toward co-occurrence. The *P* values are determined by a Fisher's exact test, $P < 0.05$ (**A**). A plot showing the relationship between CAP1, CAP2 mRNA abundance and CNA in the CAP1, CAP2 gene in tumors from the selected cancer study (**B**, **C**). The "x"s indicate individual tumors, and the circles indicate tumors with missense mutations. Homdel, homozygously deleted; Hetloss, heterozygously deleted; Diploid, two alleles present; Gain, low-level gene amplification event; Amp, high-level gene amplification event; Mutated, nonsynonymous mutation; Normal, no mutation or CNA present.

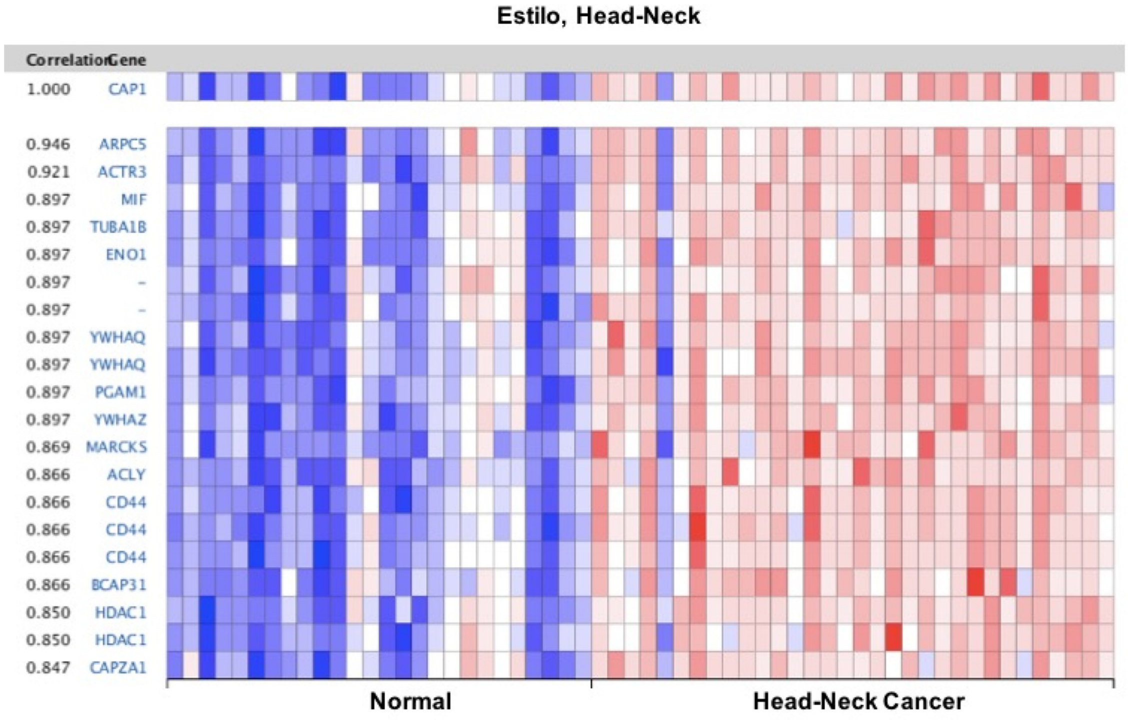

Supplementary Figure 10: CAP1 genes in Head-Neck cancer. CAP1 is coexpressed with the indicated 52 genes across a panel of 32 Head-Neck and 26 normal Head-Neck samples. Bar length represented the significance and negative logarithm of enrichment *p*-value.

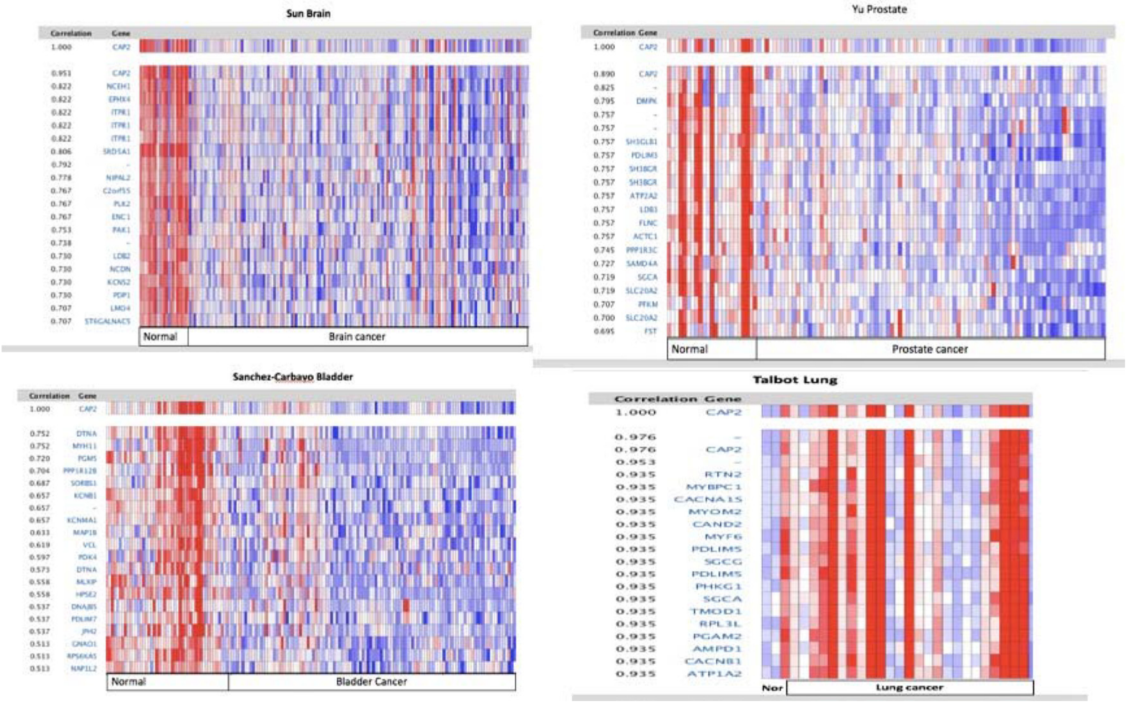

Supplementary Figure 11: CAP2 genes in Brain, Prostate, Bladder and Lung cancer. CAP2 is coexpressed with the indicated 513 genes across a panel of 157 Brain and 23 normal Brain samples, 521 genes across a panel of 89 Prostate and 23 normal prostate samples, 1051 genes across a panel of 109 Bladder and 48 normal bladder samples, 35 genes across a panel of 65 Lung and 28 normal lung samples. Bar length represented the significance and negative logarithm of enrichment *p*-value.

A

Reactome Pathways	Axon guidance<br>Developmental Biology<br>Role of Abl in Robo-Slit signaling<br>Signaling by Robo receptor
GO Terms	actin binding<br>activation of adenylate cyclase activity<br>cell morphogenesis<br>cytoskeleton organization<br>establishment or maintenance of cell polarity<br>neuronal postsynaptic density<br>plasma membrane<br>protein binding<br>signal transduction

B

Reactome Pathways	Axon guidance<br>Developmental Biology<br>Hemostasis<br>Platelet activation, signaling and aggregation<br>Platelet degranulation
GO Terms	actin binding<br>actin cytoskeleton organization<br>activation of adenylate cyclase activity<br>ameboidal-type cell migration<br>cell morphogenesis

Supplementary Figure 12: The potential pathway and GO processes were visualized by ICGC Data Portal with CAP1(**A**) and CAP2 (**B**).

A

CAP1 (chr1:40278841-40310908)

Cancer Subset	In Peak?	Nearest Peak	#Genes in Peak	Q-value	Frequency of Amplification		
					Overall	Focal	High-level
all_cancers	No	chr1:39915914-40235249	7	1.28E-11	0.1709	0.0613	0.0147
Lung NSC	Yes	chr1:39594268-40694964	20	5.03E-6	0.2524	0.0887	0.0232
Ovarian	Yes	chr1:39297788-43447561	52	0.00167	0.2524	0.1942	0.0291
all_hematologic	Yes	chr1:36669764-47094569	146	0.0501	0.03	0.0086	0.0043
Acute lymphoblastic leukemia	Yes	chr1:35626744-47094569	166	0.074	0.0153	0.0128	0.0051
Breast	Yes	chr1:32459181-68120069	333	0.311	0.2305	0.1317	0.0329
all_epithelial	No	chr1:39927775-40235249	6	1.74E-13	0.22	0.087	0.0212
all_lung	No	chr1:39927775-40164339	5	9.26E-13	0.27	0.1021	0.0323
Lung SC	No	chr1:40092174-40164339	1	1.65E-8	0.575	0.325	0.2
Esophageal squamous	No	chr1:118282114-118807006	2	1.0	0.3409	0.0682	0.0
Myeloproliferative disorder	No	No peak on chromosome	0	1.0	0.014	0.0	0.0
all_neural	No	chr1:97553627-143814988	166	1.0	0.1106	0.0184	0.0046
Colorectal	No	chr1:143657867-170995569	459	1.0	0.1366	0.0373	0.0062
Glioma	No	chr1:202625619-202824693	3	1.0	0.2683	0.0488	0.0
Hepatocellular	No	chr1:145279761-156420150	266	1.0	0.0579	0.0083	0.0
Medulloblastoma	No	No peak on chromosome	0	1.0	0.0859	0.0078	0.0
Melanoma	No	chr1:120041950-120160448	3	1.0	0.2523	0.018	0.0
Prostate	No	chr1:143798352-207231108	682	1.0	0.087	0.0326	0.0
Renal	No	chr1:143768074-199251103	580	1.0	0.1667	0.0397	0.0

B

CAP1 (chr1:40278841-40310908)

Cancer Subset	In Peak?	Nearest Peak	#Genes in Peak	Q-value	Frequency of Deletion		
					Overall	Focal	High-level
all_cancers	No	chr1:26397970-27516104	24	1.0	0.1185	0.0147	0.0
Myeloproliferative disorder	Yes	chr1:742429-45006976	586	0.0255	0.0279	0.0279	0.0
Medulloblastoma	Yes	chr1:742429-58517991	700	0.773	0.0703	0.0156	0.0
Esophageal squamous	Yes	chr1:742429-41746845	538	1.0	0.1591	0.0227	0.0
Lung SC	Yes	chr1:742429-247135059	1967	1.0	0.1	0.0	0.0
Ovarian	Yes	chr1:19832656-117038289	761	1.0	0.1165	0.0194	0.0
Prostate	Yes	chr1:742429-201213710	1639	1.0	0.1304	0.0435	0.0
Hepatocellular	No	chr1:53737764-86641362	141	0.635	0.157	0.0248	0.0
Acute lymphoblastic leukemia	No	chr1:47429728-47652865	5	1.0	0.0281	0.0	0.0
Breast	No	chr1:7843010-34898808	348	1.0	0.2263	0.0288	0.0
Colorectal	No	chr1:3756301-31327853	325	1.0	0.1366	0.0124	0.0
Glioma	No	chr1:3802708-6239708	11	1.0	0.122	0.0	0.0
Lung NSC	No	chr1:6627943-33011301	340	1.0	0.131	0.0109	0.0
Melanoma	No	chr1:68738027-143600822	281	1.0	0.2252	0.018	0.0
Renal	No	chr1:742429-29081584	390	1.0	0.1111	0.0	0.0
all_epithelial	No	chr1:26771712-27516104	13	1.0	0.1517	0.016	0.0
all_hematologic	No	chr1:47429728-47652865	5	1.0	0.03	0.01	0.0
all_neural	No	chr1:24404731-38247234	195	1.0	0.1198	0.0092	0.0
all_lung	No	chr1:9746371-31610219	284	1.0	0.1292	0.0103	0.0

C

CAP2 (chr6:17501714-17666002)

Cancer Subset	In Peak?	Nearest Peak	#Genes in Peak	Q-value	Frequency of Amplification		
					Overall	Focal	High-level
all_cancers	Yes	chr6:110391-23626250	95	1.0	0.2162	0.0351	0.0166
Breast	Yes	chr6:8665316-22771501	50	0.0333	0.3333	0.1358	0.0535
Ovarian	Yes	chr6:459991-23453081	93	0.0386	0.301	0.1553	0.068
all_epithelial	Yes	chr6:110391-24080365	95	0.699	0.2816	0.0492	0.0217
Esophageal squamous	No	No peak on chromosome	0	1.0	0.3409	0.0455	0.0
Lung SC	No	No peak on chromosome	0	1.0	0.475	0.075	0.025
Myeloproliferative disorder	No	No peak on chromosome	0	1.0	0.0	0.0	0.0
all_neural	No	No peak on chromosome	0	1.0	0.129	0.0184	0.0092
Acute lymphoblastic leukemia	No	chr6:135573332-135713904	1	1.0	0.1074	0.0026	0.0
Colorectal	No	chr6:37990758-43040693	47	1.0	0.1615	0.0124	0.0
Glioma	No	No peak on chromosome	0	1.0	0.0488	0.0	0.0
Hepatocellular	No	chr6:43788317-44414096	11	1.0	0.3884	0.0	0.0413
Lung NSC	No	chr6:43634442-44304556	15	1.0	0.3083	0.0382	0.0068
Medulloblastoma	No	No peak on chromosome	0	1.0	0.1172	0.0156	0.0078
Melanoma	No	No peak on chromosome	0	1.0	0.4505	0.018	0.0631
Prostate	No	No peak on chromosome	0	1.0	0.1087	0.0217	0.0109
Renal	No	chr6:54352087-55392731	3	1.0	0.0397	0.0	0.0
all_hematologic	No	chr6:110391-16887930	77	1.0	0.0701	0.0029	0.0057
all_lung	No	chr6:43430209-44597785	25	1.0	0.3178	0.0401	0.0078

D

CAP2 (chr6:17501714-17666002)

Cancer Subset	In Peak?	Nearest Peak	#Genes in Peak	Q-value	Frequency of Deletion		
					Overall	Focal	High-level
all_cancers	No	chr6:1553630-2553994	2	1.0	0.1115	0.024	0.0
all_hematologic	Yes	chr6:110391-20509888	90	0.0466	0.0272	0.0172	0.0
Lung NSC	Yes	chr6:110391-20509888	90	1.0	0.1282	0.0232	0.0
Ovarian	Yes	chr6:110391-170747902	999	1.0	0.068	0.0097	0.0
Prostate	Yes	chr6:110391-26509417	151	1.0	0.0652	0.0109	0.0
Acute lymphoblastic leukemia	No	chr6:26306895-26337127	11	1.0	0.0051	0.0026	0.0
Breast	No	chr6:110391-1254918	5	1.0	0.1193	0.0165	0.0
Colorectal	No	chr6:1553630-2553994	2	1.0	0.1491	0.0124	0.0
Esophageal squamous	No	chr6:161626932-170747902	34	1.0	0.1591	0.0455	0.0
Glioma	No	chr6:162959624-165587554	2	1.0	0.1707	0.0244	0.0
Hepatocellular	No	chr6:101022997-101938033	1	1.0	0.0826	0.0	0.0
Lung SC	No	chr6:110391-17472693	78	1.0	0.075	0.0	0.0
Medulloblastoma	No	No peak on chromosome	0	1.0	0.0938	0.0078	0.0
Melanoma	No	chr6:161626932-163103553	1	1.0	0.0811	0.0	0.0
Myeloproliferative disorder	No	No peak on chromosome	0	1.0	0.0093	0.0	0.0
Renal	No	No peak on chromosome	0	1.0	0.2063	0.0079	0.0
all_epithelial	No	chr6:1553630-2553994	2	1.0	0.1387	0.0248	0.0
all_neural	No	chr6:161626932-166488961	5	1.0	0.1014	0.0184	0.0
all_lung	No	chr6:110391-15624264	73	1.0	0.1253	0.022	0.0

Supplementary Figure 13: Examples of Tumorscape data analysis and visualization. (**A–B**) shows that CAP1 was most frequently amplified and deletion in lung, ovarian, breast, prostate, leukemia cancer; (**C–D**) shows that CAP2 was most frequently amplified and deletion in lung, ovarian, prostate, breast cancer; The *q*-value represents the likelihood of a random occurrence of the specific amplification/deletion that is calculated based on the background copy number variation. The table depicts a list of cancers in which the representative gene (*CAP1, CAP2)* is located in or near the frequently amplified region (orange and yellow rows, respectively).
